# Open database analysis of scaling and spatio-temporal properties of power grid frequencies

**DOI:** 10.1038/s41467-020-19732-7

**Published:** 2020-12-11

**Authors:** Leonardo Rydin Gorjão, Richard Jumar, Heiko Maass, Veit Hagenmeyer, G. Cigdem Yalcin, Johannes Kruse, Marc Timme, Christian Beck, Dirk Witthaut, Benjamin Schäfer

**Affiliations:** 1 Forschungszentrum Jülich, Institute for Energy and Climate Research-Systems Analysis and Technology Evaluation (IEK-STE), Jülich, Germany; 2 Institute for Theoretical Physics, University of Cologne, Köln, Germany; 3 Karlsruhe Institute of Technology, Institute for Automation and Applied Informatics, Eggenstein-Leopoldshafen, Germany; 4 Department of Physics, Istanbul University, 34134 Vezneciler, Istanbul, Turkey; 5 Network Dynamics, Center for Advancing Electronics Dresden (cfaed) and Institute for Theoretical Physics, Technical University of Dresden, Dresden, Germany; 6 School of Mathematical Sciences, Queen Mary University of London, London, UK

**Keywords:** Energy modelling, Complex networks

## Abstract

The electrical energy system has attracted much attention from an increasingly diverse research community. Many theoretical predictions have been made, from scaling laws of fluctuations to propagation velocities of disturbances. However, to validate any theory, empirical data from large-scale power systems are necessary but are rarely shared openly. Here, we analyse an open database of measurements of electric power grid frequencies across 17 locations in 12 synchronous areas on three continents. The power grid frequency is of particular interest, as it indicates the balance of supply and demand and carries information on deterministic, stochastic, and control influences. We perform a broad analysis of the recorded data, compare different synchronous areas and validate a previously conjectured scaling law. Furthermore, we show how fluctuations change from local independent oscillations to a homogeneous bulk behaviour. Overall, the presented open database and analyses constitute a step towards more shared, collaborative energy research.

## Abstract (web summary)

Power grid frequencies mirror the state of the grid. Here, Rydin Gorjão et al. analyse measurements of power grid frequencies across areas and continents and uncover scaling laws of their fluctuations and spatio-temporal dynamics, which could aid the design, operation and control of power systems.

## Introduction

The energy system, and in particular the electricity system, is undergoing rapid changes due to the introduction of renewable energy sources, to mitigate climate change^1^. To cope with these changes new policies and technologies are proposed^2,3^, and a range of business models are implemented in various energy systems across the world^4^. New concepts, such as smart grids^5^, flexumers^6^, or prosumers^7^, are developed and tested in pilot regions. Still, studies rarely systematically compare different approaches, data, or regions, in part because freely available research data are lacking.

The frequency of the electricity grids is a key quantity to monitor, as it follows the dynamics of consumption and generation: a surplus of generation, e.g., due to an abundance of wind feed-in, directly translates into an increased frequency. Vice versa, a shortage of power, e.g., due to a sudden increase in demand, leads to a dropping frequency. Many control actions monitor and stabilise the power-grid frequency when necessary, so that it remains close to its reference value of 50 or 60 Hz^8^. Implementing renewable energy generators introduces additional fluctuations, as wind or photo-voltaic generation may vary rapidly on various timescales^9–11^ and reduces the overall inertia available in the grid^12^. These fluctuations pose new research questions on how to design and stabilise fully renewable power systems in the future.

Analysis and modelling of the power-grid frequency and its statistics and complex dynamics have become increasingly popular in the interdisciplinary community, attracting much attention from mathematicians and physicists as well. Studies have investigated, e.g., different dynamical models^13–15^, compared centralised vs. decentralised topologies^16–18^, investigated the effect of fluctuations on the grid’s stability^19,20^, or how fluctuations propagate^21,22^. Further research proposed real-time pricing schemes^23^, optimised the placement of (virtual) inertia^24,25^, or investigated cascading failures in power grids^26–29^. However, these theoretical findings or predictions are rarely connected with real data of multiple existing power grids.

In addition to the need raised by theoretical models from the physics and mathematics community, there is also a great need for open databases and analyses from an engineering perspective. Although there exist databases of frequency time series, such as GridEye/FNET^30^ or GridRadar (https://gridradar.net/), these databases are not open, which limits their value for the research community. In particular, different scientists with access to selected, individual types of data only, from grid frequencies to electricity prices, demand and consumption dynamics, cannot combine their data with these databases, thereby hindering to study more complex questions, such as the impact of price dynamics or demand control on system stability.

Hence, open empirical data are necessary to validate theoretical predictions, adjust models, and apply new data analysis methods. Furthermore, a direct comparison of different existing power grids would be very helpful when designing future systems that include high shares of wind energy, as they are already implemented in the Nordic grid, or by moving towards liberal markets, such as the one in Continental Europe. Proposals of creating small autonomous cells, i.e., dividing large synchronous areas into microgrids^31^ should be evaluated by comparing synchronous power grids of different size to estimate fluctuation and stability risks. In addition, cascading failures, spreading of perturbations, and other analyses of spatial properties of the power system may be evaluated by recording and analysing the frequency at multiple measurement sites.

In this study, we present an analysis of an open database for power-grid frequency measurements^32^ recorded with an Electrical Data Recorder (EDR) across multiple synchronous areas^33,34^. Details on how the recordings were made are described in ref. ^32^, whereas we focus on an initial analysis and interpretation of the recordings, which are publicly available (https://osf.io/by5hu/). First, we discuss the statistical properties of the various synchronous areas and observe a trend of decreasing fluctuation amplitudes for larger power systems. Next, we provide a detailed analysis of a synchronised wide-area measurement carried out in Continental Europe. We perform a detailed analysis showing that short time fluctuations are independent, whereas long timescale trends are highly correlated throughout the network. We extract the precise timescales on which the power-grid frequency transitions from localised to bulk dynamics. Finally, we extract inter-area oscillations emerging in the Continental European (CE) area. Overall, by establishing this database and performing a first analysis, we demonstrate the value of a data-driven analysis in an interdisciplinary context.

## Results

### Data overview

**Fig. 1:**
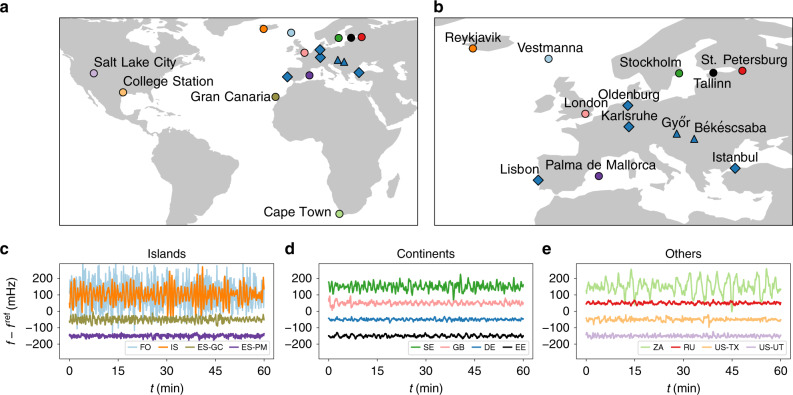
Overview of available frequency data. **a** Different locations in Europe, Africa, and Northern America at which frequency measurements were taken. Australia and large parts of Asia are not displayed, as there were no measurements recorded. **b** Zoom of the European region (excluding Gran Canaria) with all locations labelled. Circles indicate measurement sites where single measurements for several days were taken, diamonds mark the four locations where we performed GPS-synchronised measurements, and triangles mark sites for which we received additional data. **c**–**e** Frequency trajectories display very different characteristics. We plot 1 h extracts of the deviations from the reference frequency of *f*^ref^ = 50 Hz (or 60 Hz for the US power grids), which are offset from the zero mean to improve readability. Panels **c**–**e** and following plots abbreviate the measurement sites using the ISO 3166 code for each country and each location is assigned a colour code, as in the maps in **a** and **b**. For more details on the data acquisition and measurement locations, see Supplementary Note 1 and ref. ^32^. Maps were created using Python 3 and geoplots.

We recorded power-grid frequency time series using a Global Positioning System (GPS)-synchronised frequency acquisition device called EDR^33,34^, providing similar data as a Phasor Measurement Unit would. Recordings were taken at local power sockets, which have been shown to give similar measurement results as that of monitoring the transmission grid with GPS time stamps^35^ (see also ref. ^32^ for details on the data acquisition and a description of the open database). In addition, we received a 1-week measurement from the Hungarian TSO for the two cities Békéscsaba and Győr. We marked the locations of the measurement locations on a geographic map in Fig. 1a, b. Still, many more synchronous areas in the Americas, Asia, Africa, and Australia should be covered in the future.

To gain a first impression of the frequency dynamics, we visualise frequency trajectories in different synchronous areas and note quite a distinct behaviour (see Fig. 1c–e). We refer to each measurement by the country or state in which it was recorded (see also Supplementary Note 1). We group the measurements into (European) continental areas, (European) islands, and other (non-European) regions, which are also mostly continental. Most islands, such as Gran Canaria (ES-GC), Faroe Islands (FO), and Iceland (IS), but also South Africa (ZA), display large deviations from the reference frequency, whereas the continental areas, such as the Baltic (EE) and Continental European areas (DE), as well as the measurements taken in the United States (US-UT and US-TX) and Russia (RU), stay close to the reference frequency. There are still more differences within each group: e.g., the dynamics in ES-GC and ZA are much more regular then the very erratic jumps of the frequency over time observable in the FO and IS areas. Finally, we do not observe any qualitative difference between 50 and 60 Hz areas (right), when adjusting for the different reference frequency. It is noteworthy that some of the synchronous areas considered here are indeed coupled via high-voltage direct current (HVDC) lines but still possess independent synchronous behaviour. Specifically, the British (GB), Continental (DE), Baltic (EE), and Nordic (SE) European areas, as well as Mallorca (ES-PM), are connected in this way. The HVDC connection of Mallorca towards Continental Europe might be the reason it displays overall smaller deviations than the FO or IS areas, which cannot access another large synchronous area for balance.

**Fig. 2:**
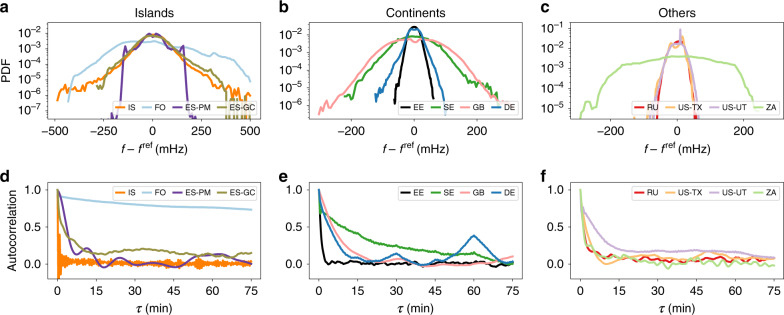
Heterogeneity in power-grid statistics. Both histograms and autocorrelation functions display very distinct features between the different synchronous areas. **a**–**c** Histograms of the different synchronous areas provide insight on heavy tails but also the different scales of the fluctuations. We visualise the empirical probability distributions of the various areas by histograms on a logarithmic scale. **d**–**f** The complex autocorrelation decay reveals distinct timescales in the different grids. We compute the autocorrelation of each area for a time lag of up to 75 min.

Let us quantify the different statistics in a more systematic way by investigating distributions (histograms) and autocorrelation functions of the various areas. The distributions contain important information of how likely deviations from the reference frequency are, how large typical deviations are (width of the distribution), whether fluctuations are Gaussian (histogram displays an inverted parabola in log-scale), and whether they are skewed (asymmetric distribution). Analysing the distributions (histograms) of the individual synchronous areas (Fig. 2a–c), we note that the islands tend to exhibit broader and more heavy-tailed distributions than the larger continental areas. Still, there are considerable differences within each group. For example, we observe a larger standard deviation (SD) and thereby broader distribution in the Nordic (SE) and British (GB) areas compared to Continental Europe (DE), which is in agreement with earlier studies^36,37^. Some distributions, such as those for Russia (RU) or the Baltic grid (EE), do show approximately Gaussian characteristics, whereas for several other areas, such as ES-GC and IS, they exhibit a high kurtosis (*κ*^IS^ ≈ 7, as compared to *κ* = 3 for a Gaussian), i.e., heavy tails, and thereby a high probability for large frequency deviations. We provide a more detailed analysis of the first statistical moments, i.e., SD *σ*, skewness *β*, and kurtosis *κ* in Supplementary Note 1.

Complementary to the aggregated statistics observable in histograms, the autocorrelation function contains information on intrinsic timescales of the observed stochastic process (see Fig. 2d–f). For simple stochastic processes such as Ornstein–Uhlenbeck processes, we would expect an exponential decay $\exp (-\gamma \tau )$ of the autocorrelation with some damping constant *γ*^38^. Although most synchronous areas do show an approximately exponential decay, the decay constants vary widely. For example, the autocorrelation of the Icelandic data (IS) rapidly drops to zero, whereas the autocorrelation of the Nordic grid (SE) has an initial sharp drop, followed by a very slow decay. Other grids, such as the Faroe Islands (FO) or the Western Interconnection (US-UT) do show a slow decay, indicating long-lasting correlations, induced, e.g., via correlated noise. Finally, regular power dispatch actions every 15  min are clearly observable in the Continental European (DE), British (GB), and also the Mallorcan (ES-PM) grids, consistent with earlier findings^36,37,39^.

In conclusion, we see that histograms are a good indicator of how heavy-tailed the frequency distributions are, whereas the autocorrelation function reveals information on regular patterns and long-term correlations. These correlations are likely connected to market activity or regulatory action, demand and generation mixture, and other aspects specific to each synchronous area. Instead of going deep into individual comparisons, let us search for general applicable scaling laws instead.

### Scaling of individual grids

For the first time, we have the opportunity to analyse numerous synchronous areas of different size, ranging from Continental Europe with a yearly power generation of about 3000 TWh^40^ and a population of hundreds of millions to the Faroe Islands with a population of only tens of thousands. These various areas allow us to test a previously conjectured scaling law^36^ of fluctuation amplitudes given as $\epsilon \sim 1/\sqrt{N}$, i.e., the aggregated noise amplitude *ϵ* in a synchronous area should decrease like the square root of the effective size of the area.

To derive this scaling relation, we formulate a stochastic differential equation of the aggregated frequency dynamics. A basic model, also known as the aggregated swing equation^41,42^, is given as:

1 $$M\frac{\,\text{d}}{\text{d}\,t}\bar{\omega }\left(t\right)=-M\gamma \bar{\omega }\left(t\right)+\Delta P(t),$$

with bulk angular velocity $\bar{\omega }$, total inertia of a region *M*, power imbalance Δ*P*(*t*), and effective damping to inertia ratio *γ*, which also comprises primary control. The bulk angular velocity is the scaled deviation of the frequency from the reference: $\bar{\omega }=2\pi \ \left(f-{f}^{\text{ref}}\right)$ and Δ*P*(*t*) effectively represents noise acting on the system with mean $\langle\Delta P(t)\rangle =0$, as generation and load are balanced on average. A simple scaling law for the frequency variability can be derived if the short-term power fluctuations at each grid node are assumed to be Gaussian. If the grid has *N* nodes with identical noise amplitudes, the SD of the power imbalance scales as:

2 $${\sigma }_{\Delta P} \sim \sqrt{N}.$$

At the same time, the total inertia typically scales linearly with the size of the grid, i.e., *M* ~ *N*. As a result, the amplitude of the total noise acting on the angular velocity dynamics scales as:

3 $$\epsilon \sim \frac{1}{M}{\sigma }_{\Delta P} \sim \frac{1}{\sqrt{N}}.$$

A more detailed derivation is provided in Supplementary Note 2 and discussed in refs. ^36,37^. In addition, a technical discussion of extracting the aggregated noise amplitude is presented in ref. ^43^. We note that the scaling law has to be modified if the noise at the nodes is not Gaussian^36^.

**Fig. 3:**
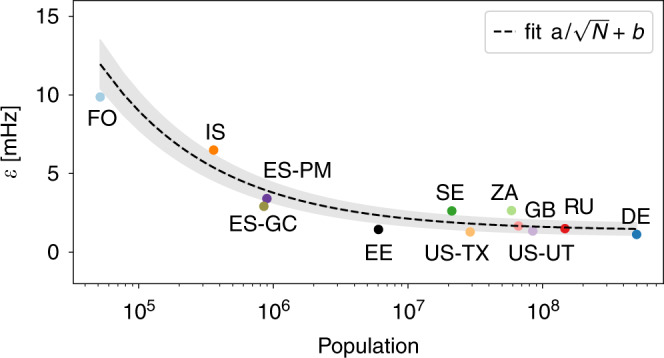
The noise tends to decrease with an increasing size of the synchronous area until it saturates. We plot the extracted noise amplitude *ϵ* compared to the logarithm of the population in a given synchronous area. The population size serves as a proxy for the total generation and consumption of that area, as data on the size of the power grids is not commonly available. The shaded area is the SD of the *ϵ* estimation.

To verify the proposed scaling law in Eq. (3), we approximate the number of nodes *N* by the population of an area, as generation data are not available for all synchronous areas, and population and generation tend to be approximately proportional^40^. We utilise the population size as a proxy for size of the grid *N*. Indeed, we note that the aggregated noise amplitude *ϵ* does approximately decay with the inverse square root of the population size, as predicted (see Fig. 3). At a certain size, the noise saturates. The deviations from the prediction, such as by ZA and IS, are likely caused by different local control mechanisms, or non-Gaussian noise distributions, which we focus on in the next section. Interestingly, although FO and ES-PM do display non-Gaussian probability density functions, they follow the proposed scaling law. Why this is the case and how a fully non-Gaussian scaling law could capture this even better remain open questions for future work. Still, we observe a decay of the noise, approximately following the prediction over four orders of magnitude.

### Increment analysis

In the previous section, we approximated the noise acting on each synchronous area as Gaussian to derive an approximate scaling law. In the following, we want to go beyond this simplification and investigate the rich short time statistics present in each synchronous area. We will see in particular how non-Gaussian distributions clearly emerge on the timescale of a few seconds.

This short timescale is investigated via increments Δ*f*_*τ*_. The increment of a frequency time series is computed as the difference of two values of the frequency with a time lag *τ*:

4 $$\Delta {f}_{\tau }=f(t+\tau )-f(t).$$

An analysis of Δ*f*_*τ*_ provides information on how the time series changes from one time lag *τ* to the next. On a short timescale of *τ* ≈ 1 s, the increments can be used as a proxy for the noise *ϵ* acting on the system (see also Supplementary Note 2).

**Fig. 4:**
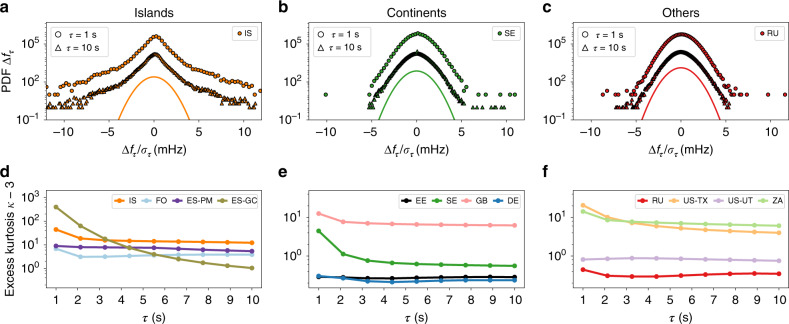
Increment analysis reveals non-Gaussian characteristics dominantly in islands. **a**–**c** We display histograms of the increments Δ*f*_*τ*_ for the lag values *τ* = 1, 10 s for selected areas. The curves are shifted for visibility and compared to a Gaussian distribution as reference. **a** Iceland (IS) displays clear deviations from Gaussianity, even for larger increments *τ*. **b** The Nordic area (SE) displays a non-Gaussian distribution for *τ* = 1 s, but approaches a Gaussian distribution for larger delays *τ*. **c** Russia (RU) has a Gaussian increment distribution for all lags *τ*. **d**–**f** We plot the excess kurtosis *κ* − 3 for the different examined power-grid frequency recordings on a log-scale. We observe a non-vanishing intermittency in Gran Canaria (ES-GC), Iceland (IS), Faroe Islands (FO), Mallorca (ES-PM), Britain (GB), Texas (US-TX), and South Africa (ZA). In contrast, the increments’ distribution of the Baltic (EE), Continental Europe (DE), Nordic (SE), Russia (RU) synchronous areas, and the Western Interconnection (US-UT) approach a Gaussian distribution. See also Fig. 5 for an illustration how increments are computed from a trajectory.

The increments for a Wiener process, an often used reference stochastic process, are Gaussian regardless of the lag *τ*^38^. However, for many real-world time series, ranging from heart beats^44^ and turbulence to solar and wind generation^9^, we observe non-Gaussian distributions for small lags *τ*. For many such processes with non-Gaussian increments, the probability distribution functions of the increments tend to approach Gaussian distributions for larger increments^9^. We observe a similar behaviour for the frequency statistics (see Fig. 4). The Nordic area (SE) displays deviations from Gaussianity for small lags *τ* but approximates a Gaussian distribution for larger *τ*. The Russian area (RU) even starts out with an almost Gaussian increment distributions. Contrary, the Icelandic area (IS) shows clear deviations from a Gaussian distribution for all lags *τ* investigated here. Still, for larger lags, the pronounced tails flatten and the increment distribution slowly approaches a Gaussian distribution. The non-Gaussian increments on a short timescale point to non-Gaussian driving forces, e.g., in terms of generation or demand fluctuations acting on the power grid.

To investigate the deviations of the frequency increments from Gaussian properties, we utilise the excess kurtosis *κ* − 3 of the distribution. As the kurtosis *κ*, the normalised fourth moment of a distribution, is *κ* = 3 for a Gaussian distribution, a positive excess kurtosis points to heavy tails of the distribution.

Computing the excess kurtosis *κ* − 3 for all our data sets, we observe variable degrees of deviation across the various synchronous areas (Fig. 4). In some areas, the intermittent behaviour of the increments Δ*f*_*τ*_ is subdued and the overall distribution approaches a Gaussian distribution (in EE, DE, SE, RUS, and US-UT), i.e., the excess kurtosis *κ* − 3 becomes very small (≲10^0^). In contrast, all islands as well as GB, US-TX, and ZA display large and non-vanishing intermittent behaviour, with a large excess kurtosis (~10^1^…10^2^). IS and ES-GC show impressive deviations from Gaussianity, which require detailed modelling in the future.

We summarise that smaller regions tend to display more intermittency in their increments than larger regions, again consistent with findings on the scaling of the aggregated noise amplitude *ϵ* (Fig. 3). Furthermore, we observe that increment distributions tend to approach Gaussian distributions for larger increments, as expected^9^, but with distinct time horizons that depend on the grid area. For most of the islands the excess kurtosis remains high even for lags of 10 s. In contrast, in most areas of continental size, the excess kurtosis is very small already for lags larger than 1 s. Very interesting is also the following observation: non-Gaussian distributions in the aggregated frequency statistics (Fig. 2) are not necessarily linked with non-Gaussian increments. For example in Continental Europe (DE), we observe Gaussian increments but a non-Gaussian aggregated distribution. The deviation from Gaussianity in the aggregated distribution, e.g., in terms of frequent extreme events, is likely explained by the external drivers, such as market activities^45^. Finally, the analysis presented here extends previous increment analyses^22,46^, which only considered increments of less than a second (*τ* < 1 s), whereas we observe relevant non-Gaussian behaviour for larger increments (*τ* ≥ 1 s). We further analyse the differences between aggregated kurtosis and increment kurtosis in Supplementary Note 1, and discuss Castaing’s model^47^ and superstatistics^48^ as more theoretical approaches towards increment analysis in Supplementary Note 3.

### Correlated dynamics within one area

**Fig. 5:**
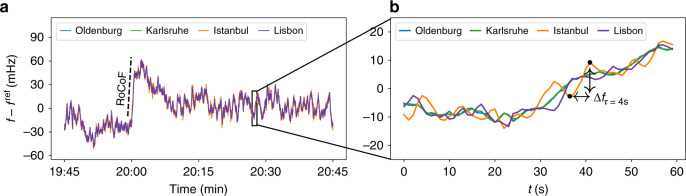
Synchronised measurements within the Continental European (CE) synchronous area differ on the short timescale. We show a 1 h frequency trajectory recorded at four different sites in the CE area: Oldenburg, Karlsruhe, Lisbon, and Istanbul (**a**, **b**). We further illustrate the RoCoF (rate of change of frequency) as the slope of the frequency every hour and the increment statistics Δ*f*_*τ*_ as the frequency difference between two points with time lag *τ*. For clarity, we do not include the two Hungarian measurement sites here, which produce similar results.

Moving away from comparing individual synchronous areas, we use GPS-synchronised measurements at multiple locations within the same synchronous area and the CE area, marked as diamonds and triangles, respectively, in Fig. 1. These measurements reveal that the frequency at different locations is almost identical on long timescales but differs on shorter timescales (see Fig. 5). Although the trajectories of the two German locations, Oldenburg and Karlsruhe, are almost identical, there are visible oscillations between the frequency values recorded in Central Europe (Karlsruhe) compared to the values recorded in the peripheries (Istanbul and Lisbon).

**Fig. 6:**
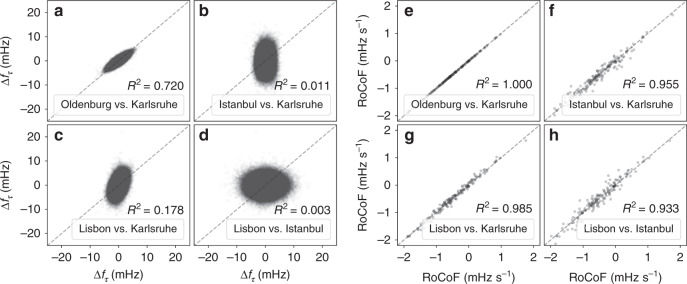
From localised fluctuations to bulk behaviour. From left to right, we move our focus from short timescales (increments) to long timescales (RoCoF). **a**–**d** Short time increments are mostly independent. We compute the increment statistics Δ*f*_*τ*_ = *f*(*t* + *τ*) − *f*(*t*) for the increment time *τ* = 1 s at the four sites in Continental Europe. The squared correlation coefficient *R*^2^ is rounded to 4 digits. See also Supplementary Note 4 for larger lags *τ* and more details. **e**–**h** Correlations at the long timescale. We record the estimated rate of change of frequency (RoCoF) d*f*/d*t* every 60 min for all four grid locations. In the scatter plots, each point represents the RoCoF or increment value Δ*f*_*τ*_ computed at two different locations at the same time *t*.

Let us quantify this by analysing the time series at the timescale of 1 s and hours (see Fig. 6). Increments Δ*f*_*τ*_, as also introduced above, reveal the short-term variability of a time series. In addition, we measure the long-term correlations on a timescale of hours by determining the rate of change of frequency (RoCoF). The RoCoF is the temporal derivative of the frequency and thereby very similar to increments. However, here it has a very different meaning, as we evaluate it only at every full hour and take into account several data points (see ref. ^37^ and Methods). Thereby, the RoCoF mirrors the hourly power dispatch^49^ and gives a good indication of long-term dynamics and deterministic external forcing. In the next section, we will also investigate the intermediate timescale of several seconds and inter-area oscillations.

Short timescale dynamics, as determined by frequency increments Δ*f*_*τ*_, are almost independent on the timescale of *τ* = 1 s (see Fig. 6a–d). We generate scatter plots of the increment value Δ*f*_*τ*_(*t*) at the same time *t* at two different locations. If the increments are always identical, all points should lie on a straight line with slope 1. If the increments are completely uncorrelated, we would expect a circle or an ellipse aligned with one axis. Indeed, the increments taken at the same time for Oldenburg and Karlsruhe are highly correlated and almost always identical, i.e., the points in a scatter plots follow a narrow tilted ellipse (Fig. 6a). Moving geographically further away from Karlsruhe, the increments of Istanbul (Fig. 6b) are completely uncorrelated with those recorded in Karlsruhe, i.e., large frequency jumps in Istanbul may take place at the same time as small jumps happen in Karlsruhe. A similar picture of uncorrelated increments emerges when comparing Lisbon and Istanbul (Fig. 6d), whereas Lisbon vs. Karlsruhe displays some small correlation (Fig. 6c). At the two peripheral locations, Lisbon and Istanbul, the increment distributions are much wider, i.e., larger jumps on a short timescale are much more common in Istanbul and Lisbon than they are in Karlsruhe. For larger lags *τ* > 1 s, the increments between all pairs become more correlated (see Supplementary Note 4).

Let us move to longer timescales. At the 60 min time stamps, power is dispatched in the CE grid to match the current demand, leading to a sudden surge in the frequency^37,39,49^. Interestingly, the frequency dynamics at the different grid sites are very similar, i.e., the deterministic event of the power dispatch is seen unambiguously everywhere in the synchronous area, almost regardless of distance (see Fig. 6e–h). All locations closely follow the same trajectory on the 1 h timescale. This is reflected in highly correlated RoCoF values, with a particularly good match between Oldenburg and Karlsruhe, and a linear regression coefficient of at least *R*^2^ ≥ 0.93 for all pairs (Fig. 6e–h).

**Fig. 7:**
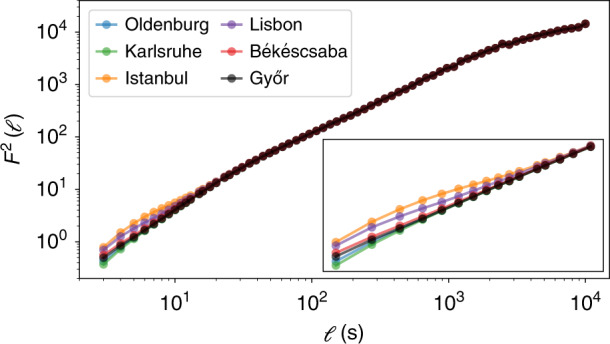
Detrended Fluctuation Analysis (DFA) connects short and long timescales. We perform a DFA^65^, with order *m* = 1, in accordance with ref. ^64^, and plot the fluctuation function *F*^2^(*ℓ*) as a function of the time window length *ℓ*. The inset magnifies the values for *ℓ* ∈ {10^0^…2 × 10^1^}. The lines connect data points to each other to guide the eye.

We combine these different timescales in a single detrended fluctuation analysis (DFA), where we also integrate the two Hungarian locations (see Fig. 7). At short timescales, the DFA results differ for the six locations, while starting at the timescale of *t* ~ 10^1^ s, the four curves coincide. For the timescale of 1 s, all locations are subject to different fluctuations, with Istanbul and Lisbon displaying the largest values of the fluctuation function. This is coherent with results of the increment analysis, where Istanbul and Lisbon have the broadest increment distributions (Fig. 6a–d). Moving to longer timescales of tens or hundreds of seconds, we observe a coincidence of the fluctuation function. This coincidence, i.e., identical behaviour for large timescales is in good agreement with the highly correlated RoCoF results (Fig. 6e–h). We may also interpret this change from short-term and localised dynamics to long-term and bulk behaviour as a change from stochastic to deterministic dynamics, i.e., the random fluctuations are localised and take place on a short timescale, whereas the deterministic dispatch actions and overall trends penetrate the whole grid on a long timescale. See also Methods and Supplementary Note 5 for details on the DFA methodology.

### Spatio-temporal dynamics

Next, let us investigate the spatio-temporal aspect of the synchronised measurements. We connect the transition from local fluctuations towards bulk behaviour with the geographical distance of the measurement points, complementing earlier analysis based on voltage angles^50,51^. We determine the typical time-to-bulk, i.e., the time necessary so that the dynamics at a given node approximates the bulk behaviour. To this end, we choose Karlsruhe, Germany, as our reference, which is very central within the CE synchronous area. The choice of the reference does not qualitatively change the results. For each of the remaining five locations, we compute the relative DFA function:

5 $$\eta (\ell )=\frac{{{F}^{2}}^{\text{Location}}(\ell )-{{F}^{2}}^{\text{Karlsruhe}}(\ell )}{{{F}^{2}}^{\text{Karlsruhe}}(\ell )}$$

with respect to Karlsruhe and ask, when does this difference drop below 0.1 (or 10%), i.e., when are the fluctuation at each location almost indistinguishable from the ones in Karlsruhe?

**Fig. 8:**
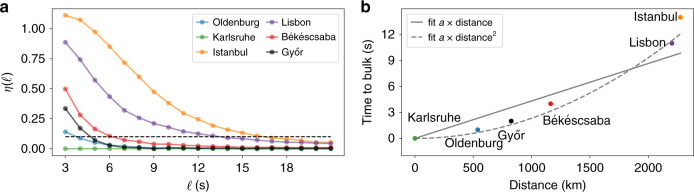
The time-to-bulk increases with distance. **a** The relative DFA function *η*(*ℓ*) with Karlsruhe as reference; we determine the time-to-bulk as the time when this value reaches 0.1 (dashed line) (see also Eq. (5)). **b** We plot the so-extracted time-to-bulk vs. the distance from Karlsruhe and provide a simple fit for the first five points (i.e., excluding Istanbul, as it clearly behaves differently). We obtain a value for the linear fit *a* = 5.2 × 10^−1^ s km^−1^, see Methods for details on the distance.

The further apart two locations are, the later they reach the bulk behaviour, i.e., the larger their time-to-bulk (see Fig. 8). This observation can be intuitively understood: two sites in close geographical vicinity are typically tightly coupled and can be synchronised by their neighbours, whereas sites far away have to stabilise on their own. Our time-to-bulk analysis quantifies this intuition. We consider both a linear and a quadratic fit. A linear dependence is expected if the bulk behaviour is realised by coupling via the shortest available path. In contrast, if the propagation is following a diffusive pattern via multiple independent paths, we would expect a quadratic dependence of the time with respect to the distance. Indeed, the quadratic fit, following diffusive coupling, is a much better fit than a linear one, as indicated by a lower root-mean-squared-error 0.5, compared to 1.2 s in the linear case. Using the newly obtained fits, we find that a location only 100 km from Karlsruhe will have to independently stabilise fluctuations on the scale of 0.5–1 s and will then closely synchronise with the dynamics in Karlsruhe (our bulk reference). In contrast, a site 1000 km away has to stabilise already for about 3–5 s before it is fully integrated in the bulk. This gives additional guidance for the control within large synchronous areas, in particular for remote and weakly coupled sites. Clearly, these first estimates demonstrate that further research is necessary to validate and adjust spatio-temporal models of the power grid^21^.

### Principal component analysis

So far, we have focused on when and how the localised fluctuations transition into a bulk behaviour. During this transition, on the intermediate timescale of about 5 s, we observe another phenomenon: ‘Inter-area oscillations’, i.e., oscillations between sites in different geographical areas far apart but still within one synchronous area. Different methods are available to extract spatial inter-area modes, ranging from Empirical Mode Decomposition^52^ to nonlinear Koopman modes^53^. Here we use a principal component analysis (PCA)^54^, which was already introduced to power systems when analysing inter-area modes and identifying coherent regions^55^. A PCA separates the aggregated dynamics observed in the full system into ordered principal components, which we interpret as oscillation modes. Ideally, we can explain most of the observed dynamics of the full system by interpreting a few dominant modes. Each of these modes contains information of which geographical sites are involved in the modes dynamics, similar to an eigenvector. Typical behaviour includes a translational dynamics of all sites (the eigenvector with entries 1 everywhere) or distinct oscillations between individual sites (an eigenvector with entry 1 at one site and −1 at another site).

**Fig. 9:**
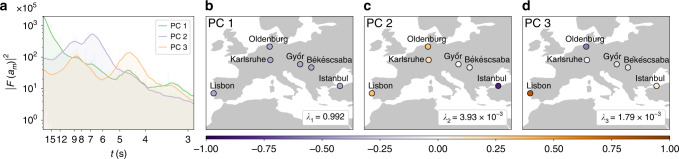
Principal component analysis (PCA) of frequency recordings reveals inter-areas oscillations. **a** In Continental Europe, the squared Fourier amplitudes ${\left|{\mathcal{F}}\left({a}_{m}(t)\right)\right|}^{2}$ of the three dominant principal components (PCs) exhibits typical period lengths of *t* ≈ 7 s and *t* ≈ 4.5 s. **b** While the first spatial mode (PC1) corresponds to the bulk behaviour of the frequency and explains already *λ*_1_ = 99.2% of the total variance, the second (PC2) and third (PC3) mode reveal asynchronous inter-area modes (**c**, **d**). We refer to Supplementary Note 6 for details on the method and the results.

Indeed, applying a PCA to the synchronised measurements in CE, we can capture almost the entire dynamics with just three modes (see Fig. 9). In Fig. 9a, we provide the squared Fourier amplitudes of each mode and in Fig. 9b–d we visualise the first three modes geographically. These three modes already explain the largest shares *λ*_m_ of the total variance (see Supplementary Note 6 for the remaining modes and more details). The first mode (PC1) explains *λ*_1_ ≈ 99.2% of the variance and represents the synchronous bulk behaviour of the frequency. The second (PC2) and third (PC3) mode correspond to asynchronous inter-area modes. They contribute much less to the total variance due to their small amplitude (cf. Fig. 5). In PC2 (Fig. 9c), Western Europe forms a coherent region that is in phase opposition to Istanbul (East–West dipole), whereas in PC3 (Fig.  9d), Lisbon and Istanbul swing in opposition to Oldenburg (North–South dipole). Similar results were found in an earlier theoretical study of the CE area, which also revealed global inter-area modes with dipole structures^56^.

The temporal dynamics of the spatial modes exhibit typical frequencies of inter-area oscillations. Figure 9a shows the squared Fourier amplitudes |*F*(*a*_*m*_(*t*))|^2^ of the spatial modes. The components PC2 and PC3 have their largest peaks at *t* ≈ 7 s and *t* ≈ 4.5 s, which are the periods of these inter-area modes. These periods correspond well to the typical periods of inter-area oscillations, which are reported to be 1.25–8 s^57^. On larger timescales *t* > 12 s, the amplitudes |*F*(*a*_*m*_(*t*))|^2^ of the inter-area modes drop below the values of PC1. Thus, the frequency dynamics is dominated by the bulk behaviour again, which is consistent with the estimated time-to-bulk of 12–15 s (Fig. 8).

## Discussion

In this study, we have presented a detailed analysis of a recently published open database of power-grid frequency measurements^32^. We have compared various independent synchronous areas, from small regions, such as the FO and ES-PM areas, to large synchronous areas, such as the Western Interconnection in North America and the CE grid, spanning areas with only tens of thousand customers to those with hundreds of millions. Especially the smaller areas tend to show a larger volatility in terms of aggregated noise but also increment intermittency, such as IS and ES-GC. We have complemented this analysis of independent grids by GPS-synchronised measurements within the CE power grid, revealing high correlations of the frequency at long timescales but mostly independent dynamics on fluctuation-dominated short timescales. Compared to other studies applying synchronised, wide-area measurements, such as FNET/Grideye in the US^30^ or evaluations from IS^58^, the data we analysed here is freely available for further research^32^.

The comparison of different synchronous areas gives us a solid foundation to test previously conjectured scaling laws of fluctuations in power grids with their size^36^, helps us to develop synthetic models^37^, or predict the frequency^59^ of small grids, such as microgrids. Furthermore, aggregating standardised measurements from different areas, we can compare countries with high shares of renewables (high hydro generation in Iceland or the Nordic area) with areas with almost no renewable generation (Mallorca) to learn how they influence the frequency dynamics and thereby the power-grid stability. Similarly, this comparison also gives insights on how different market structures impact the frequency statistics and stability of a power grid.

Our results on the spatial dependencies in the CE synchronous area are also highly relevant for the operation of power grids and other research in the field. The observations that the long-term behaviour is almost identical throughout the synchronous area but short time fluctuations differ, are in agreement with earlier theoretical findings^21^. Based on the DFA results (Figs. 7 and 8), we provide a quantitative estimate that at least for the CE area already at timescales of about 10 s, we observe an almost uniform bulk behaviour, even for locations thousands of kilometres apart. This bulk behaviour emerges much faster when locations are closer to one another.

In the regime of resonant behaviour^21^, we observe inter-area oscillations with period lengths of *t* = 7 s and *t* = 4.5 s, which we extract using a PCA. These timescales agree well with frequencies of inter-area oscillations reported in other studies in Europe^56,57,60^ but also in the United States^61^. However, we notice that the timescales separating bulk, resonance and local behaviour are different than the authors in a theoretical work^21^ assumed. There, local fluctuations were described for the 0.1 s timescale and bulk dynamics already started at times between about 2 and 5 s. This raises the question on how these timescales depend on the size and the dynamics of the power grid under consideration. Finally, we note that the PCA is a prime example for a model-free and data-driven analysis that leads to better understanding.

Our observation of frequency increments being independent on timescales of 1 s is consistent with earlier studies^46^. For Continental Europe, we find that 1 s increments are correlated at small distances (below 500 km), but independent at locations far apart. On timescales of 1 s and below, we cannot observe global inter-area modes anymore. Instead, we expect local fluctuations that quickly decay with distance to their origin^21,22^, which is consistent with our findings. The distribution of these short-term fluctuation was reported to exhibit a strongly non-Gaussian distribution when subject to intermittent wind power feed-in^46^. In agreement with these results, the non-Gaussian effects vanish on timescales above 1 s in our recordings from Continental Europe. However, in other, particularly smaller, synchronous areas we even observe heavy-tailed increment distributions on timescales up to 10 s. This is likely related to the grid size and control regulations, although a detailed explanation still remains open.

In this study, we connect the mathematics and physics communities with the engineering community, by providing potent data analysis tools from the theoretical side and then connecting these findings in the practical domain of power-grid dynamics without the use of an explicit model. Both the data analysis and its interpretation could be very useful for the operation of individual grids. Our insights for the scaling could be used to improve control mechanisms, such as demand side management^62^, whereas our spreading insights give further indications about how fast cascading failures will spread throughout the power grid^28^. Several grid operators and other researchers have likely recorded power-grid frequency time series at many more grid locations than we could provide in this single study. All such recordings from different sources should be combined to enable more comparisons between the dynamics of synchronous areas of different sizes and under different conditions. The database studied here^32^ may offer a valuable starting point for such endeavours.

As data are still only scarcely available, there remain many open questions: can we systematically determine a propagation velocity of disturbances through the grid and compare these with theoretical predictions^21,25,63^? Can we identify other time series influencing the power-grid frequency dynamics and quantify their correlation such as hydro power plants in the Nordic area or demand of aluminium plants in IC? Can we extract the impact of market activities on the frequency dynamics in all synchronous areas? From a statistical modelling perspective, it would be interesting to investigate the scaling of higher moments, i.e., skewness and kurtosis, with time lag and size in more detail. These questions constitute only a small selection from a multitude that an open database may help to address from a broad, interdisciplinary perspective, including engineering, mathematics, data science, time series analysis, and many other fields.

## Methods

### Data selection

We make use of the open database, described in detail in ref. ^32^, to perform all analyses presented in the main text and in Supplementary Notes 1–6. This data set contains recordings of 12 independent synchronous regions recorded between 2017 and 2020. Although some locations, such as the FO area only contain a single week of data, other regions, such as Continental Europe have been monitored for several months or years (for more details, see ref. ^32^). However, due to some technical difficulties, e.g., loss of GPS signal or unplugging the device, some measurements are not a number, i.e., ‘NaN’, and are tagged as not reliable in the database. These entries have been deleted to compute the histograms and statistical measures in Supplementary Note 1. To compute the autocorrelation function and for the analysis of the synchronised measurement in Continental Europe, we selected the longest possible trajectory without any ‘NaN’ entries. As a final note, from the available ES-GC data, we are using the March 2018 data.

### RoCoF computation

When determining the RoCoF, i.e., the time derivative of the frequency, we follow the same procedure as has been outlined in ref. ^37^: we select a short time window centred around the anticipated dispatch jumps at 60 min of about 25 s length, i.e., starting at (*X*) : 59 : 48 and lasting until (*X* + 1) : 00 : 12 for all hours *X*. Then, we fit this short frequency trajectory with a linear function *f*(*t*) = *a* + *bt*. We are not interested in the offset *a* but the value of *b* gives us the slope of the frequency changes, i.e., the time derivative of the frequency is approximately given as $\frac{\,{\text{d}}f}{{\text{d}}\,t}\approx b$.

### Detrended fluctuation analysis

To carry out the DFA we follow a similar procedure as outlined in ref. ^64^, using the package outlined in ref. ^65^. The main idea is to detrend the data and extract the most dominant timescales by measuring the scaling behaviour of the data from increasing segments of data. The commonly denoted fluctuation function *F*^2^(*ℓ*), function of the segment size *ℓ* on the time series, accounts for the variance of segmented data of increasing size. The scaling of the underlying process or processes can thus be extracted. In ref. ^64^, a detailed study of the different timescales in power-grid frequencies can be found, largely focusing on scales of about 10 s and above, whereas we put particular emphasis on the smallest timescales available, of the order of 1 s. More details are given in Supplementary Note 5.

### Time-to-bulk

To extract the time-to-bulk, seen in Fig. 8, we take the measurements of the DFA in Fig. 7 and utilise Karlsruhe as the reference for comparison. Having Karlsruhe as a reference, we compare the normalised fluctuations *η*(*ℓ*):

6 $$\eta (\ell )=\frac{{{F}^{2}}^{\text{location}}(\ell )-{{F}^{2}}^{\text{Karlsruhe}}(\ell )}{{{F}^{2}}^{\text{Karlsruhe}}(\ell )},$$

(Eq. (5) in the main text), to extract the excess fluctuation at the different locations. As there is no standard, we choose a threshold value of 10% for fluctuations at the different recordings to be identical. Once *η*(*ℓ*) drops below this threshold of 10%, the data sets are considered to be identical. In this manner, we determine the time-to-bulk as the necessary time of a recording to exhibit the same fluctuation behaviour as the reference of Karlsruhe. The distance measures taken are the geographic distances with respect to Karlsruhe, applying OpenStreet Maps https://www.openstreetmap.org/ and using the routing by Foot(OSRM). This yields the following distances from Karlsruhe: Oldenburg: 538 km, Győr: 825 km, Békéscsaba: 1163 km, Lisbon: 2203 km, Istanbul: 2276 km. The reason to use route finding by foot is that the power grid is not taking any air plane routes but is limited also to the shortest routes available in the transmission grid. These distances in the power system might be even longer where transmission line density is low. It is noteworthy that our choice of geographical distance does not apply any assumption on the underlying power-grid topology. With full (yet currently unavailable) information about all operational transmission lines, a shortest path distance on the transmission network would be an alternative^22^.

## Supplementary information

Supplementary Information

Peer Review File

## Data Availability

Frequency recordings are described in detail in ref. ^32^. An open repository containing all recordings can be accessed here: https://osf.io/by5hu/. The Hungarian TSO data are available here: https://osf.io/m43tg/. All data that support the results presented in the figures of this study are available from the authors upon reasonable request.

## Code availability

Code to produce the presented analysis and figures is available on github: https://github.com/LRydin.
