# Supplementary material for: Open database analysis of scaling and spatio-temporal properties of power grid frequencies
Source: Nat Commun. 2020 Dec 11;11:6362. doi: 10.1038/s41467-020-19732-7 (PMC7732984; doi:10.1038/s41467-020-19732-7)
Supplement: Supplementary file 1 — Supplementary Information [file 41467_2020_19732_MOESM1_ESM.pdf]

Supplementary Information accompanying the manuscript Open data base analysis of scaling and spatio-temporal properties of power grid frequencies

Leonardo Rydin Gorjão,[1,2] Richard Jumar,[3] Heiko Maass,[3] Veit Hagenmeyer,[3] G.Cigdem Yalcin,[4] Johannes Kruse,[1,2] Marc Timme,[5] Christian Beck,[6] Dirk Witthaut,[1,2] and Benjamin Schäfer[6,*]

[1] *Forschungszentrum Jülich, Institute for Energy and Climate Research - Systems Analysis and Technology Evaluation (IEK-STE), Germany*
[2] *Institute for Theoretical Physics, University of Cologne, Germany*
[3] *Karlsruhe Institute of Technology, Institute for Automation and Applied Informatics, Germany*
[4] *Department of Physics, Istanbul University, 34134, Vezneciler, Turkey*
[5] *Chair for Network Dynamics, Center for Advancing Electronics Dresden (cfaed) and Institute for Theoretical Physics, Technical University of Dresden, Germany*
[6] *School of Mathematical Sciences, Queen Mary University of London, United Kingdom*

Within this Supplementary Information, we give further details on the analysis presented in the main text. In particular, we introduce the abbreviations used to describe the various measurement sites, report the first four statistical moments of all areas. Furthermore, we provide more details on the PCA analysis, including a visualization of the modes and a comparison with the spectrum.

* b.schaefer@qmul.ac.uk

SUPPLEMENTARY NOTE 1

Details on individual measurements: Abbreviations and statistical measures

Abbreviations

The power grid frequency has been recorded in several synchronous areas across Europe and beyond. We introduce the abbreviations used when referring to the measurement sites, e.g. in plots, in Supplementary Table I: We list the town, and country where the measurement was taken and the synchronous area to which this particular device was connected. For example, the measurement taking place in Karlsruhe, Germany is abbreviated as DE (ISO 3166 for Germany) and is connected to the Continental European synchronous area (CE). When discussing the results, we will often refer to the synchronous area and name the measurement site abbreviation in parenthesis, e.g. Continental Europe (DE) is known to be influenced by market dynamics, see also [1]. Unless we are specifically interested in the short time scale, a measurement taken in one location is representative for the whole synchronous area, see also discussion on time-to-bulk in the main text. For areas that are part of a larger country, we first name the country and then specify the area further, e.g. US-UT stands for the United States of America, State Utah, which in turn is part of the Western Interconnection.

Supplementary Table I. Abbreviations of measurement locations and their connection to synchronous areas. For each country, we adopt the ISO 3166 code. The population is extracted from Wikipedia and the sources therein in March 2020.

Abbreviation	Measurement location	Synchronous area	Population
**Islands**			
IS	Reykjavík, Iceland	Iceland	360,390
FO	Vestmanna, Faroe Islands	Faroe Islands	51,783
ES-GC	Las Palmas de Gran Canaria, Canary Islands, Spain	Gran Canaria	851,231
ES-PM	Palma de Mallorca, Balearic Islands, Spain	Mallorca	896,038
**Continental**			
DE	Karlsruhe, Germany	Continental Europe (CE)	500,000,000
GB	London, United Kingdom	Great Britain (GB)	66,224,800
EE	Tallin, Estonia	Baltic	6,042,657
SE	Stockholm, Sweden	Nordic	21,180,931
**Others**			
US-UT	Salt Lake City, Utah, US	Western Interconnection	84,600,000
US-TX	College Station, Texas, US	Texas Interconnection	28,995,881
ZA	Cape Town, South Africa	South Africa	58,775,022
RU	St. Petersburg, Russia	Russia	146,745,098

Statistical measures

Let us systematically investigate the statistical properties of the various areas by computing their mean deviation from the reference μ, as well as their standard deviation σ, skewness β, and kurtosis κ in Supplementary Table II. First, we note that most of the synchronous areas are very close to their reference frequency of 50 Hz (or 60 Hz for US areas) on average. Except for Great Britain (GB) and South Africa (ZA), continental areas display a smaller standard deviation than islanded areas. For GB, we can attribute this large deviations to the very different frequency regulation framework (when compared to Continental Europe), which allows large deviations [2]. The skewness and kurtosis are more difficult to interpret. Some areas, such as Texas (US-TX) and Faroe Islands show a substantial skewness, while other areas, such as Continental Europe (DE) and Iceland (IS) display a large kurtosis $\kappa > 3$, hinting at heavy tails in the distribution.

Kurtosis of aggregated data and increment statistics Let us have a closer look at the kurtosis values. We utilise the kurtosis and its deviation from the Gaussian value of $\kappa^{\text{Gauss}} = 3$ to quantify whether a given distribution displays heavy tails and thereby deviates from a Gaussian distribution. For the aggregated statistics studied above this is relevant as it tells us how likely we will observe extreme deviations, which could lead to a curtailment of demand or a

Supplementary Table II. Statistical properties of different measurement sites. We report the mean μ as the mean deviation from the reference frequency $f - f^{\text{ref}}$, standard deviation σ, skewness β and kurtosis κ of the data.

Area	Mean μ [mHz]	Std. σ [mHz]	Skew. β	Kurtosis κ
**Islands**				
IS	0.90	55.81	-0.04	7.02
FO	-36.65	133.15	0.27	2.39
ES-GC	-0.60	61.63	0.38	5.00
ES-PM	1.32	70.13	0.06	2.62
**Continental**				
DE	0.22	18.77	0.08	3.95
GB	-0.06	62.29	0.04	2.42
EE	-0.29	12.44	-0.09	2.95
SE	0.61	48.91	0.19	4.05
**Others**				
US-UT	-0.73	18.67	-0.04	2.56
US-TX	0.20	17.79	-0.49	2.42
ZA	1.47	93.43	-0.15	2.39
RU	-1.63	16.44	-0.24	2.52

shutdown of generators. For the increments, this is of particular interest for the statistical modelling since a classical Ornstein–Uhlenbeck Process would lead to Gaussian increments [3].

A kurtosis of $\kappa > 3$ is only clearly observed in the aggregated data of IS, ES-GC, DE, and SE. In all cases, the numerous extreme events are a remarkable observation. For such a highly regulated and controlled system as the power grid to deviate so strongly so often demands an explanation. We are confident that the heavy tails at the two continental areas DE and SE are related to extreme deviations at the trading intervals [1, 4, 5]. The tails in the two islands might also be caused by market activities or arise due to a different operation by the TSO, as will be clarified in future work.

The role of the kurtosis changes when moving to increments. A large kurtosis of the increments indicates that the effective noise acting on the system does indeed not follow a Gaussian distribution, as expected in many stochastic processes. Naturally, investigating the increments of an empirical trajectory, we will observe a much more volatile behaviour than if we only observe the trajectory itself. Large jumps will be present and on a short time scale we expect extreme tails in real-world data. Some of these large jumps could be caused by renewable generators, others by fast control mechanisms or inverters. Still it is remarkable that kurtosis values of κ 10^2, are observed for IS and ES-GC in Fig. 4 of the main text. While the general trend of decreasing kurtosis with increasing time lag is in coherence with previous work on increment analysis [6, 7], further work is necessary to arrive at a full statistical description of frequency increment.

SUPPLEMENTARY NOTE 2

Scaling of fluctuations

Let us investigate whether the fluctuation in terms of regular deviations, measured by the standard deviation σ, or extreme deviations, measured by the kurtosis κ scale with the size of the power system. Intuitively, we would expect that the more generation is present in a given area, the smaller the fluctuations we observe are going to be. As a proxy of the total generation, we use the total population of a synchronous area as this information is easily available for all areas and population and total generation are approximately proportional [8]. As we can see in Supplementary Fig 1, the kurtosis κ is not a simple function of the size of a synchronous area. The occurrence of heavy tails, as measured by the kurtosis κ, depends on dispatch strategies, market regulations [9] and control requirements, which are standardised within the European Network of Transmission System Operators for Electricity (ENTSO-E) [2], leading to very similar values of the kurtosis in most synchronous areas.

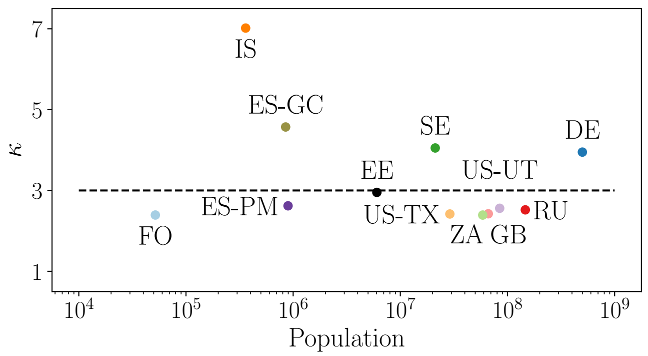

Supplementary Figure 1. Extreme fluctuation occurrence do not decrease with increasing grid size. We plot the kurtosis as a measure of heavy tails as a function of the population of a synchronous area.

In contrast, we have seen in the main text that the aggregated noise amplitude ϵ decreases approximately as the square root of the size of a synchronous area. Let us review the derivation of this relation in more detail and discuss alternative approaches to observe the scaling. We follow the arguments presented in [1]: Let us use the standard swing equation [10] to describe the synchronous frequency dynamics at each node i as

$$M_i \dot{\omega_i}(t) = -D_i \omega_i(t) + \epsilon_i \Gamma_i(t) + P_i^m + P_i^e, \tag{1}$$

where $\omega_i = f_i/(2\pi)$ is the nodal angular velocity, M_i is the inertia, D_i is the damping, $\epsilon_i \Gamma_i(t)$ is a noise term and P_i^m and P_i^e are the mechanical power generated or consumed and the transmitted electrical power respectively. Moving to the bulk description, i.e., defining the total inertia $M := \sum_{i=1}^N M_i$ and the bulk angular velocity $\bar{\omega} := \sum_{i=1}^N M_i \omega_i / M$ and assuming a constant damping to inertia ratio $\gamma = D_i/M_i$ [11] as well as balanced electrical and mechanical power on average, i.e., $\sum_{i=1}^N P_i^m = 0,\ \sum_{i=1}^N P_i^e = 0$, we obtain

$$\frac{\mathrm{d}}{\mathrm{d}t}\bar{\omega}(t) = -\gamma \bar{\omega}(t) + \frac{1}{M}\sum_{i=1}^N \epsilon_i \Gamma_i(t). \tag{2}$$

The equation from the main text is re-obtained by identifying ΔP as $\sum_{i=1}^N \epsilon_i \Gamma_i(t)$. If we assume that the noise $\Gamma_i(t)$ at each node is approximately Gaussian with zero mean and standard deviation 1 (the amplitude is included in ϵ_i), we can formulate the Fokker–Planck equation [3] of this Ornstein–Uhlenbeck process (2) as

$$\frac{\partial p}{\partial t} = \gamma \frac{\partial}{\partial \bar{\omega}}(\bar{\omega} p) + \frac{1}{2}\frac{1}{M^2}\left[\sum_{i=1}^N \epsilon_i^2\right]\frac{\partial^2 p}{\partial \bar{\omega}^2}. \tag{3}$$

The resulting probability density function $p(\bar{\omega})$ is a normal distribution with mean 0 and standard deviation

$$\sigma = \sqrt{\frac{\sum_{i=1}^N \epsilon_i^2}{\gamma M^2}}. \tag{4}$$

As an additional simplification, let us assume identical noise $\epsilon_i = \xi$ and identical inertia $M_i = m$ at all nodes, i.e., $M = Nm$. Then, the standard deviation is given as

$$\sigma = \sqrt{\frac{N\xi^2}{\gamma m^2 N^2}} = \sqrt{\frac{\xi^2}{\gamma m^2}} \frac{1}{\sqrt{N}}. \tag{5}$$

This means the standard deviation decays approximately as $\sigma \sim 1/\sqrt{N}$. An important assumption when comparing areas with Supplementary Eq. (5) is that we assume similar noise ξ, damping γ, and inertia m. While the inertia per machine will likely be similar in the different areas, the effective damping depends on the control applied and the noise on the mix of generators (nuclear, hydro, coal, wind, solar, ...) and the nature of the demand fluctuations.

To take these additional external factors into account when comparing the empirical data of the various synchronous areas, we do not compare the standard deviation several areas but their aggregated noise amplitudes ϵ. To retrieve the aggregated noise amplitude ϵ we employ a non-parametric Nadaraya–Watson estimator to extract the Kramers–Moyal moments of the underlying stochastic dynamics. For a timeseries $x(t)$, the nth Kramers–Moyal moment can be extracted via

$$M_n(x,t) = \lim_{\Delta t \to 0} \frac{1}{\Delta t} \left\langle \left(x'(t+\Delta t) - x'(t)\right)^n \middle| x'(t) = x \right\rangle, \tag{6}$$

where the averaging process is made more precise by implementing a kernel-density function $K_h(\cdot) = h^{-1}K(\cdot/h)$ with a bandwidth h. The Nadaraya–Watson estimator $W_h(x)$, at point i, is given by [12]

$$W_h(x)_i = \frac{K_h(x - x'_i)}{\sum_{j=1}^{S} K_h(x - x'_j)}, \tag{7}$$

where we take $K_h(x)$ to be an Epanechnikov function, compact in $\mathbb{R}_{[-1,1]}$, and S is the number of data points. The aggregated noise amplitude ϵ is retrieved studying the second Kramers–Moyal moment, and employing the aforementioned estimator, resulting in

$$M_2(x) = \frac{1}{S\Delta t} \sum_{i=1}^{S} W_h(x)_i [x'(t+\Delta t) - x'(t)]_i^2 = \epsilon^2, \tag{8}$$

with $\Delta t = 1s$. All Kramers–Moyal moments are easily retrievable, see Refs. [13, 14]. The so extracted aggregated noise amplitude ϵ will closely follow the empirical Fokker–Planck equation and should therefore resemble a $\epsilon \sim 1/\sqrt{N}$ decay, as any deterministic effects are filtered out.

Finally, while we expect the noise to decay with the size, it is well-justified to add a constant to our previously derived expression (5), modifying it to

$$\epsilon \sim \sigma = \frac{a}{\sqrt{N}} + b. \tag{9}$$

We add the constant b to take the effect of deadbands into account. All synchronous areas have deadbands [10], i.e. frequency ranges for which there is no (primary) control active and the frequency dynamics evolves freely. This means for a certain range $|\omega| < \omega_{\mathrm{deadband}}$ the damping to inertia ratio γ, which explicitly includes primary control, is much smaller and the frequency randomly evolves and contributes a minimum noise contribution b.

In addition to the standard deviation σ or the aggregated noise amplitude ϵ, we might also consider using a Gaussian kernel to detrend the data and only analyse the standard deviation of the detrended data. Finally, we could also consider using the standard deviation of the increments (at the smallest available time lag $\tau = 1$) as a proxy of the actual noise. We compare these different approaches in Supplementary Fig. 2: Moving from top to bottom: Taking the unfiltered standard deviation of the data (Supplementary Fig. 2 a), does give a rough trend but areas as South Africa (ZA) or Great Britain (GB) have a much larger standard deviation than we would expect from the scaling law. When we introduce the de-trending, the overall standard deviation drops (Supplementary Fig. 2 b) but the problems with GB and ZA persist. Likely this is caused by control regulations that allow larger deviations than they are allowed for example in Continental Europe. Next, we extract the aggregated noise amplitude ϵ (Supplementary Fig. 2 c) and observe a decay, well-approximating the conjectured scaling law. Finally, we note that using the standard deviation of the increments at lag $\tau = 1$s is almost identical to the aggregated noise amplitude ϵ (Supplementary Fig. 2 d). Comparing all four plots, in particularly in terms of the standard deviation of the best fit (shaded area), leads to the conclusion that panels c and d are the best descriptors. Both the extracted noise ϵ as well as the increments on the one second scale Δf_1 are good proxies of the diffusive forces acting on the synchronous area.

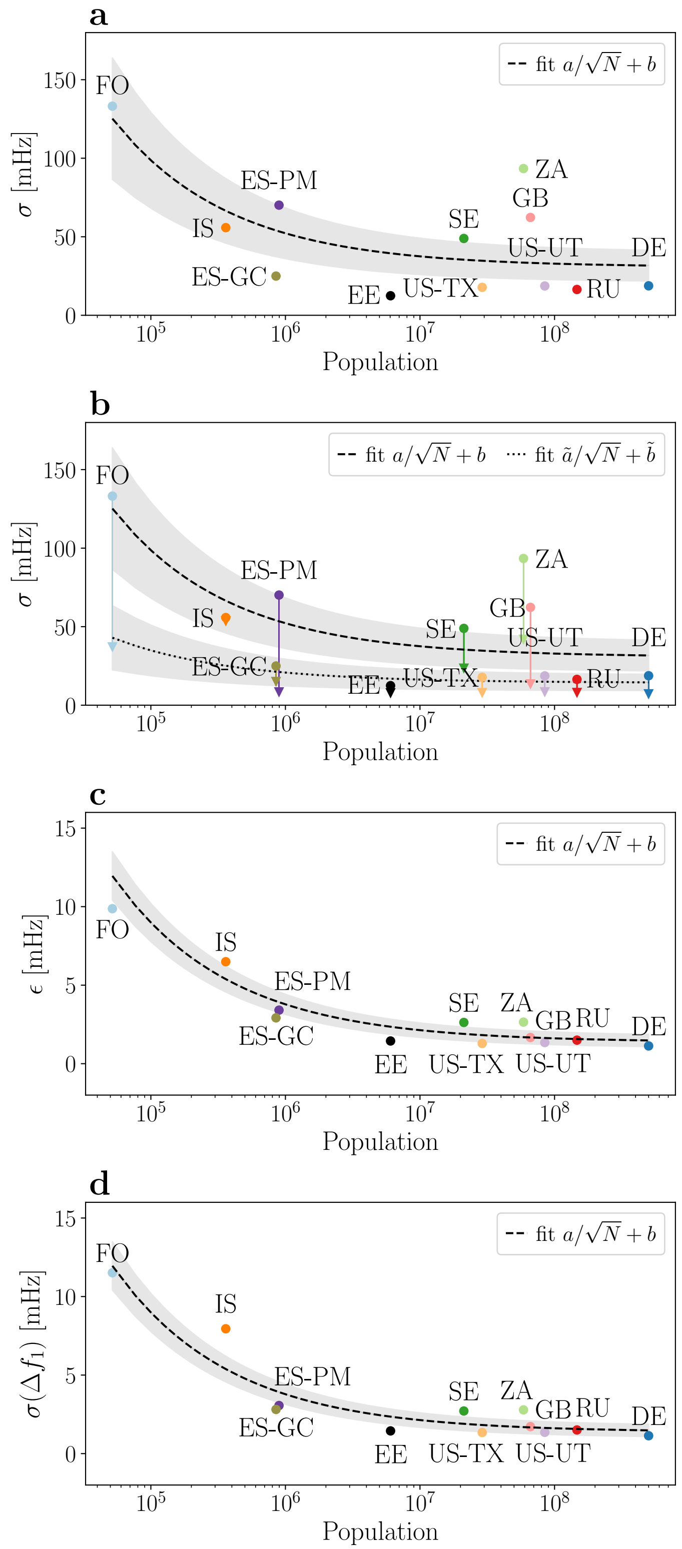

Supplementary Figure 2. Average fluctuations decrease with increasing grid size with increments Δf_1 and aggregated noise amplitude ϵ as the best descriptors. We plot the standard deviation (a), filtered standard deviation (b), the aggregated noise amplitude (c) and the standard deviation of the increments at time lag $\tau = 1$s (d). The shaded area gives the standard deviation of the fit.

SUPPLEMENTARY NOTE 3

Castaing's model for increments

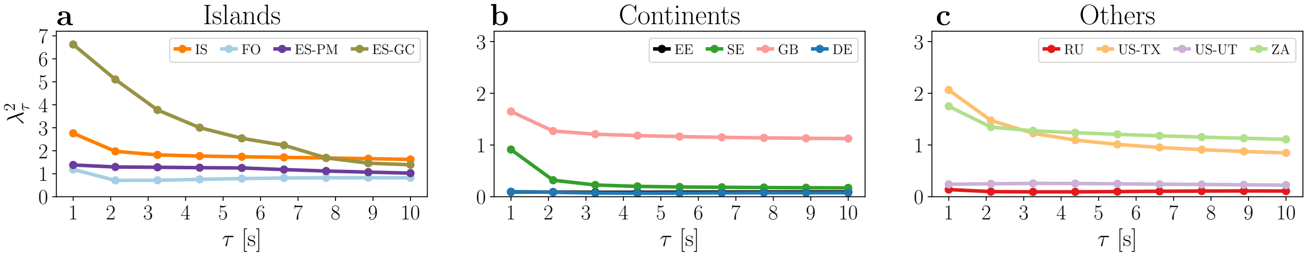

Supplementary Figure 3. Increment analysis reveals non-Gaussian characteristics, dominantly in islands. We plot the Castaing parameter λ_τ^2, given by Supplementary Eq. (14), for the different examined power grid frequency recordings. We observe a non-vanishing intermittency in Gran Canaria (ES-GC), Iceland (IS), Faroe Islands (FO), Mallorca (ES-PM), Britain (GB), Texas (US-TX) , and South Africa (ZA). In contrast, the increments' distribution of the Baltic (EE), Continental Europe (DE), Nordic (SE), Russia (RU) synchronous areas and the Western Interconnection (US-UT) approach a Gaussian distribution.

In the main text we quantified deviations from Gaussianity of the increment distributions by the use of the excess kurtosis $\kappa-3$, which should decay to zero if the distributions under consideration are Gaussian. Here, we offer a more theoretical view by using Castaing's model. This model describes the deviations of the increments' distribution from a Gaussian distribution [15–17] and has already been applied to frequency analysis [6, 7]. The increments Δf_τ with the lag τ have a non-homogenous scaling, which leads to distributions with high kurtosis, and sometimes non-zero skewness [18]. The rationale is that the process is a superposition of several subset processes with distinct scales, similar to superstatistics [19, 20]. Specifically, Castaing's model is a special case of log-normal superstatistics applied to increments. The probability density function (PDF) of the increments $p(\Delta f_\tau, \sigma_\tau)$ is a function of the widths σ_τ, given by

$$p(\Delta f_\tau, \tau) = \int_{-\infty}^{\infty} L_\lambda(\sigma_\tau) p_0(\Delta f_\tau, \sigma_\tau) d\ln\sigma_\tau, \tag{10}$$

where the underlying subset processes are assumed to have a Gaussian distribution $p_0(\Delta f_\tau, \sigma_\tau) \sim \mathcal{N}(0, \sigma_\tau)$ of some variance σ and $L_\lambda(\sigma_\tau)$ accounts thus for the scales of each superposition. This scale function $L_\lambda(\sigma_\tau)$ is conjectured to be log-normally distributed

$$L_\lambda(\sigma_\tau) = \frac{1}{\lambda_\tau\sqrt{2\pi}} \exp\left[-\frac{\ln^2(\sigma_\tau/\sigma_0)}{2\lambda_\tau^2}\right], \tag{11}$$

with λ_τ^2 being the Castaing parameter. For the case $\lambda_\tau^2 \to 0$, the distribution $L_\lambda(\sigma_\tau)$ approached a δ-distribution, and the increments Δf_τ are purely Gaussian distributed. As λ_τ^2 increases, the convolution includes more scales and the tails of the PDF of the increments enlarge. Inserting Supplementary Eq. (11) into Supplementary Eq. (10) yields the explicit PDF of the increments as

$$p(\Delta f_\tau, \tau) = \\ \frac{1}{\lambda_\tau 2\pi} \int_0^{\infty} \frac{d\sigma_\tau}{\sigma_\tau^2} \exp\left[-\frac{\Delta f_\tau^2}{2\sigma_\tau^2} - \frac{\ln^2(\sigma_\tau/\sigma_0)}{2\lambda_\tau^2}\right]. \tag{12}$$

The increments intermittency behaviour is thus solely described by the Castaing parameter λ_τ^2. Multiplying both sides of Supplementary Eq. (12) by Δf_τ^2 and integrating over $[-\infty, \infty]$, we find

$$\sigma_0^2 = \langle \Delta f_\tau^2 \rangle \exp\left[-2\lambda_\tau^2\right]. \tag{13}$$

One can now recover the Castaing parameter λ_τ^2 by extracting the fourth-order statistical moment, i.e., the kurtosis $\kappa_{\Delta f}(\tau)$, of the PDFs of Δf_τ as function of the lag τ. The Castaing parameter λ_τ^2 results in

$$\lambda_\tau^2 = \ln\left(\frac{\kappa_{\Delta f}(\tau)}{3}\right), \tag{14}$$

i.e., for a Gaussian distribution with $\kappa_{\Delta f}(\tau) = 3$ the Castaing parameter decays to 0.

To extract the Castaing parameter, we compute the increment statistics Δf_τ of a certain timeseries f_τ and calculate the kurtosis κ_τ of the PDF of the increments. Then, we take the normalised logarithm as in Supplementary Eq. (14) and do so over the desired range of increments time-lag τ.

Computing the Castaing parameter λ_τ^2 for our data, we observe very heterogeneous results between the various synchronous areas, see Supplementary Fig. 3. In some areas, the intermittent behaviour of the increments Δf_τ is subdued and the overall distribution approaches a Gaussian distribution (in EE, DE, SE, RUS, and US-TX), i.e., the Castaing parameter λ_τ^2 approaches 0. On the other hand, all islands display large and non-vanishing intermittent behaviour, as well as GB, US-TX, and ZA. Iceland (IS) but particularly Gran Canaria (ES-GC) shows impressive deviations from Gaussianity that require detailed modelling in the future.

With Castaing's model and parameter we used an alternative approach to quantify heavy tails, instead of purely using the kurtosis κ. A further advantage of Castaing's approach is that it allows us to model the increments as superimposed distributions, complementary to superstatistics of the aggregated frequency distributions [1]. An explicit increment model will be left for future work.

SUPPLEMENTARY NOTE 4

Further increment analysis

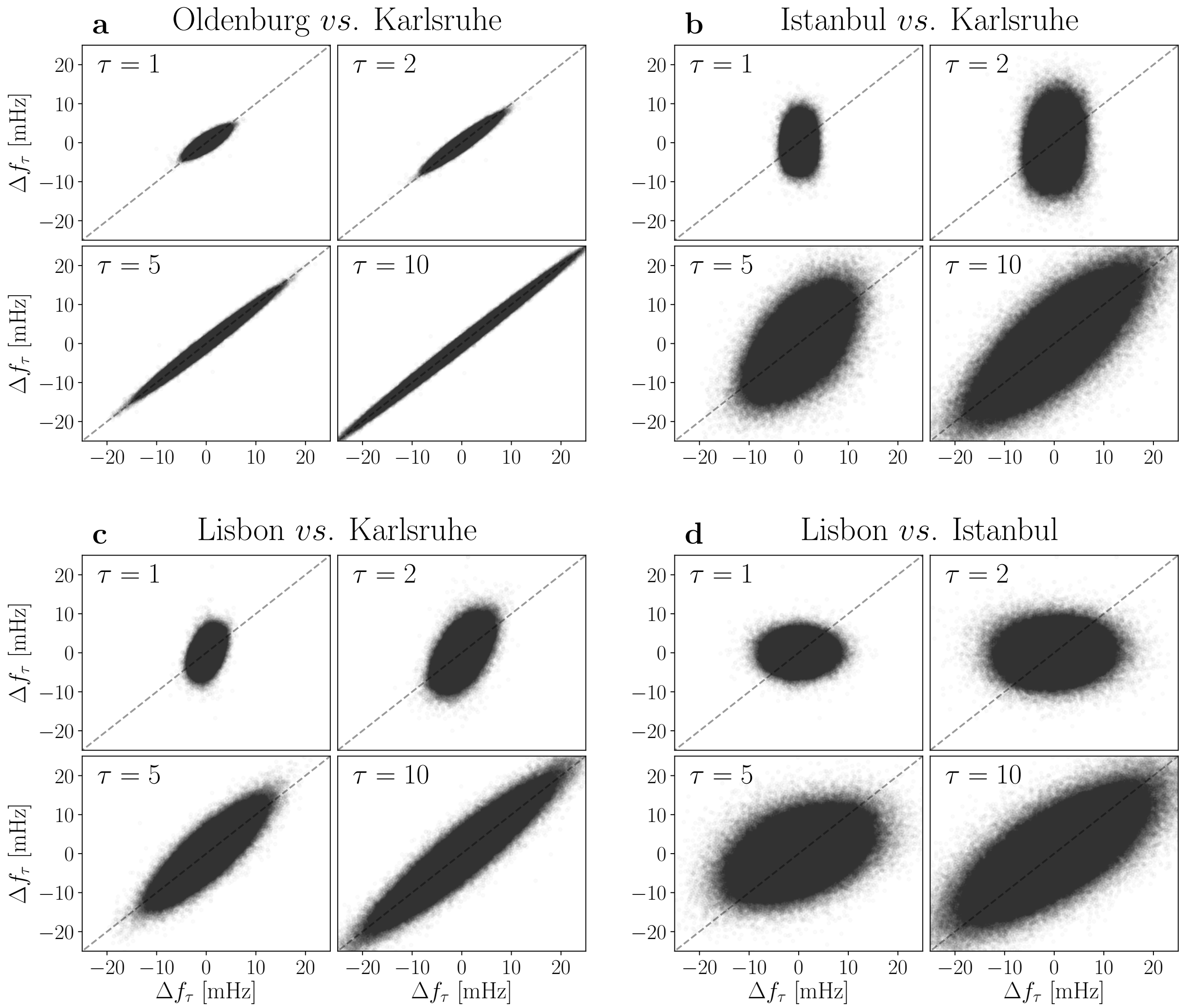

Supplementary Figure 4. Long increment lags lead to increased correlations. We repeat the increment analysis from the main text but with larger lags $1\,\mathrm{s} \leq \tau \leq 10\,\mathrm{s}$. Note that each of the $2{\times}2$ subpanels is now using four different lags τ but the overall figure still follows the same arrangement as in the main text.

We complement the increment analysis from the main text by considering larger time lags $\tau > 1\,\mathrm{s}$. In particular, we compute the increments [6, 21] $\Delta f_\tau = f(t+\tau) - f(t)$ for the four measurement sites in Continental Europe: Karlsruhe, Oldenburg, Istanbul and Lisbon. For the pairs investigated in the main text, we consider a lag $\tau = 1, 2, 5, 10$ seconds in Supplementary Fig. 4. Since the scatter plots report increments at two locations at the same time t, points on the diagonal indicate large correlations, while circles or ellipses aligned with one axis indicate no correlations. The results for Oldenburg vs. Karlsruhe are almost independent of the specific time lag τ. The magnitude of the increments increases for increasing time lag τ but almost all values follow the diagonal, indicating a high correlation on the full time scale for 1 to 10 seconds. In contrast, the increment plots involving Istanbul and Lisbon change much more with increasing lag τ as their increments on the time scale of 1 second are almost completely uncorrelated but at 5 to 10 seconds, an increasing number of points follow the diagonal, i.e., fluctuation events become correlated. Similar to the detrended fluctuation analysis (DFA) from the main text, we again observe that short time scales are independent, while we approximate a bulk description for longer time scales. This observation is also consistent with claims found e.g in [22] that high frequency fluctuations do not penetrate the grid over long distances but lower frequency fluctuations do.

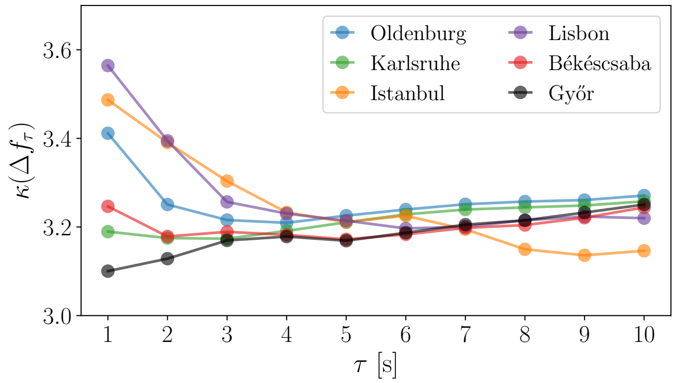

Supplementary Figure 5. The kurtosis κ of the increment statistics Δf_τ decreases with increasing lag τ. We plot the kurtosis κ of each recording site as a function of the increment lag τ.

Finally, we may further investigate how the increment distributions look like, in particular with respect to their large deviations, i.e., their heavy tails measured by the kurtosis of the increment distributions. Computing the kurtosis κ of the increment statistics Δf_τ at different lags τ, shows that the deviations from the Gaussian ($\kappa^{\text{Gaussian}} = 3$) decrease *on average*. While the kurtosis shows a small increase in Győr with increasing time lag, the kurtosis at Istanbul and Lisbon is substantially reduced, see Supplementary Fig. 5. This finding is consistent with [6], where the authors also found that long lags lead to approximately Gaussian increment statistics. Here, we go further in that we observe spatial differences already at a time resolution of 1 second, in particular for locations far away from Karlsruhe.

SUPPLEMENTARY NOTE 5

Details on Detrended Fluctuation Analysis (DFA)

Detrended Fluctuation Analysis (DFA) [23, 24] studies the fluctuation of a given process by considering increasing segments of the timeseries. Take a timeseries $X(t)$ with N elements X_i, $i = 1, 2, \dots, N$. Obtain the detrended profile of the process by defining

$$Y_i = \sum_{k=1}^{i} (X_k - \langle X \rangle) \,, \text{ for } i = 1, 2, \dots, N,$$

i.e., the cumulative sum of X subtracting the mean $\langle X \rangle$ of the data. Section the data into smaller non-overlapping segments of length s, obtaining therefore $N_s = \text{int}(N/s)$ segments. Given the total length of the data is not always a multiple of the segment's length s, discard the last points of the data. Consider the same data, apply the same procedure, but this time discard instead the first points of the data. One has now $2N_s$ segments. To each of these segments fit a polynomial y_v of order m and calculate the variance of the difference of the data to the polynomial fit

$$F^2(v, s) = \frac{1}{s} \sum_{i=1}^{s} [Y_{(v-1)s,i} - y_{v,i}]^2, \text{ for } v = 1, 2, \dots, N_s,$$

where $y_{v,i}$ is the polynomial fitting for the segment i of length v. One also has the freedom to choose the order of the polynomial fitting. This gives rise to the denotes DFA1, DFA2, ..., for the orders chosen. Notice $F^2(v, s)$ is a function of each variance of each v-segment of data and of the different s-length segments chosen. One can define the fluctuation function $F^2(s)$ by averaging each row of segments of size s

$$F^2(s) = \frac{1}{N_s} \left\{ \sum_{v=1}^{N_s} F^2(v, s) \right\}^{1/2} .$$

The inherent scaling properties of the data, if the data displays power-law correlations, can now be studied in a log-log plot of $F^2(s)$ versus s. Herein the scaling of the data obeys a power-law with exponent h as

$$F^2(s) \sim s^h,$$

where h is the *self-similarity* exponent (which may be multifractal) and relates directly to the Hurst index. The self-similarity exponent h is calculated by finding the slope of this curve in the log-log plots. For a detailed explanation of DFA, see [25]. The analysis implemented here is based on [26].

SUPPLEMENTARY NOTE 6

Principal Component Analysis

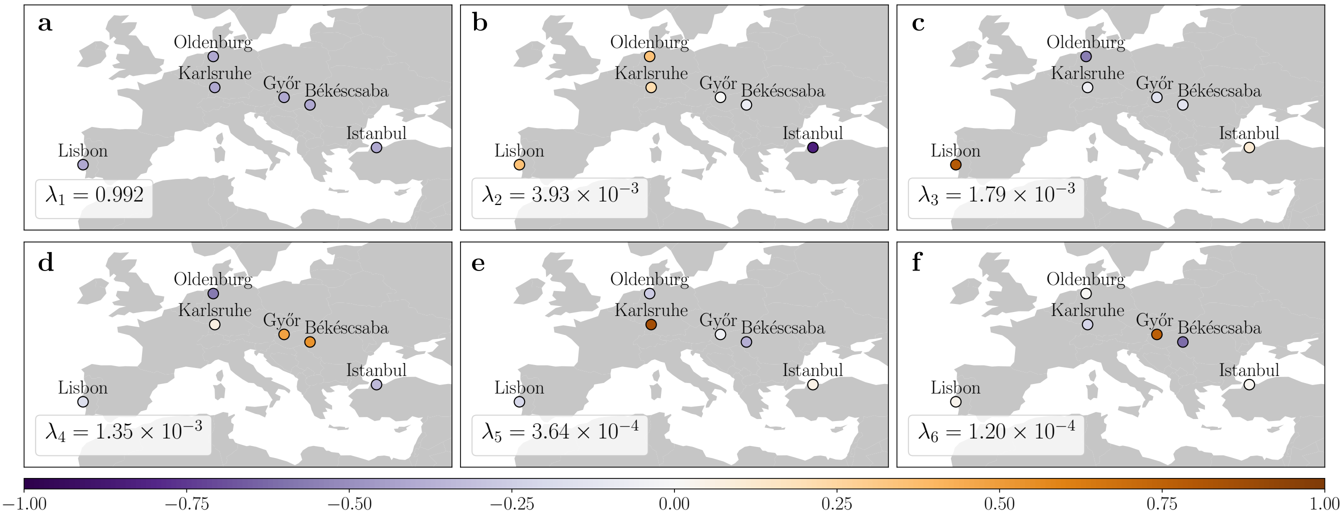

Supplementary Figure 6. The principal components reveal the spatial structure of inter-area modes. The maps show the colour-coded entries of the normalised principal components $\mathbf{f}_m$. The spatial structure corresponds to synchronous behaviour (a), East-West oscillations (b), North-South oscillations (c), Coastal-inland fluctuations (d-e), and intra-Hungarian oscillations (f). The synchronous component describes the frequency bulk behaviour and thus explains already $\lambda_1 = 99.2\%$ of the total variance.

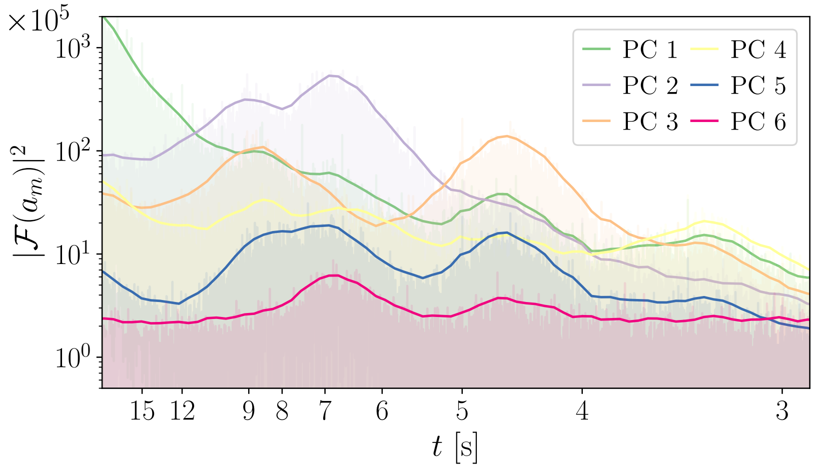

Supplementary Figure 7. The inter-area modes oscillate with a period between 3 and 9 seconds. The figure displays squared Fourier amplitudes $|\mathcal{F}(a_m(t))|^2$ of the mode amplitudes $a_m(t)$. The largest inter-area oscillations occur with a period of $t = 7$ s and $t = 4.5$ s, which corresponds well to the results in [27]. In the main text, we only discussed the first three modes as the most dominant ones.

We apply a principal component analysis (PCA) to our multivariate frequency time series in order to extract the dominant modes of inter-area oscillations. Generally, a PCA identifies the orthogonal linear subspaces (principal components) that maximise the projected variance of the data [28]. Let $\mathbf{f}(t)$ be the vector containing the frequency recordings from all six locations within Europe. The principal components are then given by the eigenvectors $\mathbf{f}_m(t)$ of the data covariance matrix. Based on the principal components, we can decompose the centred frequency recordings

into uncorrelated time series $a_m(t)$:

$$\mathbf{f}(t) = \langle \mathbf{f} \rangle + \sum_{m=1}^{N} a_m(t)\mathbf{f}_m.$$

The time series $a_m(t)$ describe the amplitude of the frequency recordings $\mathbf{f}(t)$ projected onto the m-th principal component. Their variance, i.e., the projected variance, is given by the eigenvalue $\tilde{\lambda}_m$. By rescaling this variance, we obtain the share of total variance explained by the m-th component:

$$\lambda_m = \frac{\tilde{\lambda}_m}{\sum_m \tilde{\lambda}_m}.$$

We interpret the principal components (PCs) of $\mathbf{f}(t)$ as spatial inter-area modes and the time series $a_m(t)$ as their amplitudes. Supplementary Fig. 6 displays all six modes obtained from our synchronised measurements in Continental Europe. The first mode (PC1) represents a synchronous frequency oscillation at all locations and thus corresponds to the bulk behaviour of the frequency. The other modes (PC2-PC6) display asynchronous spatial patterns and thus represent inter-area oscillations. Due to their small amplitude, these modes only explain a low portion of the variance ($\lambda_2, ..., \lambda_6 < 0.4\%$), while the bulk mode already covers $\lambda_1 \approx 99.2\%$. However, PC2 to PC6 uncover the dominant spatial structure of inter-area modes across Continental Europe. Let us analyse these modes based on their visualisation in Supplementary Fig. 6 in more detail: PC2 contains one coherent area in Western Europe that oscillates in phase opposition to Istanbul. In PC3 Lisbon and Istanbul swing in opposition to the Northern measurement locations. PC2 thus resembles an East-West dipole, while PC3 is similar to a North-South dipole. The other modes correspond to a Coast-Inland fluctuation (PC4 and PC5) and an intra-Hungarian oscillation (PC6).

To reveal the oscillation period of these modes, we analyse the power spectral density (PSD) of their amplitudes $a_m(t)$ (Supplementary Fig. 7). The East-West dipole (PC2) exhibits a main period length of $t \approx 7$ s and a smaller contribution at $t \approx 9$ s. PC3 mainly oscillates with a period pf $t \approx 4.5$ s, but there is also a distinct peak at $t \approx 9$ s, which also appears in PC2 and PC4. PC5 and PC6 mainly exhibit oscillations with a period of $t \approx 7$ s and $t \approx 4.5$ s. The PSD of bilateral differences between single measurement locations reveals the same main period lengths (Supplementary Fig. 8). Interestingly, no inter-area oscillations can be observed between Karlsruhe and Oldenburg, which could indicate well-balanced power within Germany or could be caused by the limited spatial resolution of the available 6 modes. Overall, we identify three main oscillations periods of inter-area modes with $t \approx 9$ s, $t \approx 7$ s, and $t \approx 4.5$ s, which is consistent with the typical frequency of inter-area modes [29].

The comparison to a more detailed study suggests that our principal components probably relate to overlaps of different global inter-area modes. A first indicator for this conclusion is the occurrence of multiple substantial peaks in our PSD in Supplementary Fig. 7 (e.g. for PC3). The detailed analysis in [27] revealed four dominant inter-area modes in Continental Europe with distinct period lengths. The authors describe a mode G1, which resembles a North-South dipole and oscillates with $t = 5$ s. Furthermore, they present mode T1, which represents an East-West dipole oscillating at $t = 6.7$s, and similar mode G2 with $t = 3.3$s. In mode G3, Eastern Europe, Spain, and Portugal swing in phase opposition to Central Europe with $t = 2$s. Comparing this to our results, we conclude that PC2 corresponds well to mode T1, while PC3 is similar to mode G1. However, PC3 contains another substantial oscillation with $t = 9s$. This could be an effect of aliasing due to our low Nyquist-frequency of 0.5 Hz. Modes with period lengths below 2s thus re-appear in our PSD at multiples of their oscillation period. The mode G3 could thus correspond to the peak at $t \approx 9$s in Supplementary Fig 7. Finally, PC4 contains the mode G2 among others, while PC5 and PC6 both contain the period lengths of G1 and T1. The PCA modes are thus very similar to the results in [27], but they do not isolate inter-area modes with single distinct periods.

The overlap of different (linear) oscillation modes in our PCA results can have different reasons. Our low spatial resolution could make it impossible to retain the spatial modes identified in an earlier study [27]. On the other hand, a linear separation of the inter-area modes through a PCA and a Fourier decomposition could generally fail due to the non-linearity of power system dynamics. Finally, our time resolution of one second could lead to aliasing and the incorrect reconstruction of inter-area modes. In the future, a higher spatial and temporal resolution of frequency recordings would be necessary to further investigate these effects.

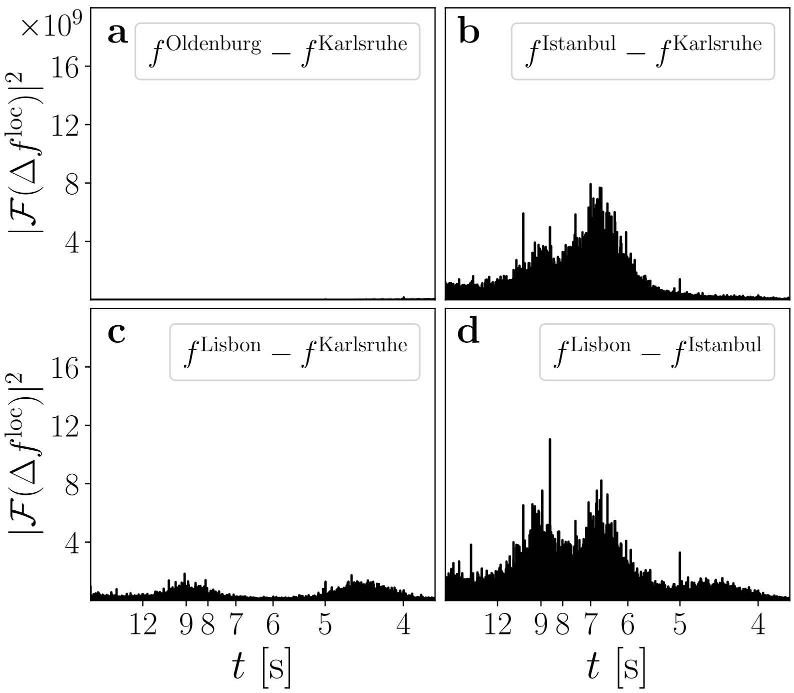

Supplementary Figure 8. Inter-area oscillations between different grid sites. We perform a spectral analysis of the frequency differences by computing the spectrum of the frequency difference Δf^{loc} between two sides, e.g. in (e): $\Delta f^{\text{loc}} = f^{\text{Oldenburg}} - f^{\text{Karlsruhe}}$.

SUPPLEMENTARY REFERENCES

[1] Schäfer, B., Beck, C., Aihara, K., Witthaut, D. & Timme, M. Non-Gaussian power grid frequency fluctuations characterized by Lévy-stable laws and superstatistics. *Nature Energy* **3**, 119–126 (2018).

[2] ENTSO-E. Network code on requirements for grid connection applicable to all generators (rfg). `https://www.entsoe.eu/major-projects/network-code-development/requirements-for-generators/` (2013).

[3] Gardiner, C. W. *Handbook of Stochastic Methods: for Physics, Chemistry and the Natural Sciences* (Springer, 1985).

[4] Rydin Gorjão, L. *et al.* Data-driven model of the power-grid frequency dynamics. *IEEE Access* **8**, 43082–43097 (2020).

[5] Schäfer, B., Timme, M. & Witthaut, D. Isolating the impact of trading on grid frequency fluctuations. In *2018 IEEE PES Innovative Smart Grid Technologies Conference Europe (ISGT-Europe)*, 1–5 (IEEE, 2018).

[6] Hähne, H., Schottler, J., Wächter, M., Peinke, J. & Kamps, O. The footprint of atmospheric turbulence in power grid frequency measurements. *Europhysics Letters* **121**, 30001 (2018).

[7] Hähne, H., Schmietendorf, K., Tamrakar, S., Peinke, J. & Kettemann, S. Propagation of wind-power-induced fluctuations in power grids. *Physical Review E* **99**, 050301 (2019).

[8] ENTSO-E. Statistical factsheet 2018. `https://docstore.entsoe.eu/Documents/Publications/Statistics/Factsheet/entsoe_sfs2018_web.pdf` (2018).

[9] Weißbach, T. & Welfonder, E. High Frequency Deviations within the European Power System–Origins and Proposals for Improvement. *VGB powertech* **89**, 26 (2009).

[10] Machowski, J., Bialek, J. & Bumby, J. *Power System Dynamics: Stability and Control* (John Wiley & Sons, Chichester, 2011).

[11] Weixelbraun, M., Renner, H., Schmaranz, R. & Marketz, M. Dynamic simulation of a 110-kv-network during grid restoration and in islanded operation. In *20th International Conference and Exhibition on Electricity Distribution-Part 1, 2009*, 1–4 (IET, 2009).

[12] Lamouroux, D. & Lehnertz, K. Kernel-based regression of drift and diffusion coefficients of stochastic processes. *Physics Letters A* **373**, 3507–3512 (2009).

[13] Rydin Gorjão, L. & Meirinhos, F. kramersmoyal: Kramers–Moyal coefficients for stochastic processes. *Journal of Open Source Software* **4**, 1693 (2019).

[14] Rinn, P., Lind, P. G., Wächter, M. & Peinke, J. The Langevin approach: An R package for modeling Markov processes.

Journal of Open Research Software **4**, e34 (2016).

[15] Castaing, B., Gagne, Y. & Hopfinger, E. Velocity probability density functions of high Reynolds number turbulence. *Physica D: Nonlinear Phenomena* **46**, 177–200 (1990).

[16] Castaing, B. Scalar intermittency in the variational theory of turbulence. *Physica D: Nonlinear Phenomena* **73**, 31–37 (1994).

[17] Castaing, B. The temperature of turbulent flows. *Journal de Physique II* **6**, 105–114 (1996).

[18] Tabar, M. R. R. *The Friedrich–Peinke Approach to Reconstruction of Dynamical Equation for Time Series: Complexity in View of Stochastic Processes*, 143–164 (Springer International Publishing, Cham, 2019).

[19] Beck, C. & Cohen, E. G. D. Superstatistics. *Physica A* **322**, 267–275 (2003).

[20] Beck, C., Cohen, E. G. D. & Swinney, H. L. From time series to superstatistics. *Physical Review E* **72**, 056133 (2005).

[21] Anvari, M. *et al.* Short term fluctuations of wind and solar power systems. *New Journal of Physics* **18**, 063027 (2016).

[22] Zhang, X., Hallerberg, S., Matthiae, M., Witthaut, D. & Timme, M. Fluctuation-induced distributed resonances in oscillatory networks. *Science Advances* **5**, eaav1027 (2019).

[23] Peng, C.-K. *et al.* Mosaic organization of DNA nucleotides. *Physical Review E* **49**, 1685–1689 (1994).

[24] Kantelhardt, J. W. *et al.* Multifractal detrended fluctuation analysis of nonstationary time series. *Physica A* **316**, 87–114 (2002).

[25] Ihlen, E. Introduction to Multifractal Detrended Fluctuation Analysis in Matlab. *Frontiers in Physiology* **3**, 141 (2012).

[26] Rydin Gorjão, L. MFDFA: Multifractal Detrended Fluctuation Analysis in Python. `https://zenodo.org/record/3625759` (2020).

[27] Grebe, E., Kabouris, J., Lopez Barba, S., Sattinger, W. & Winter, W. Low frequency oscillations in the interconnected system of Continental Europe. In *IEEE PES General Meeting*, 1–7 (IEEE, Minneapolis, MN, 2010).

[28] Bishop, C. M. *Pattern Recognition and Machine Learning* (Springer, New York, 2007), 1 edn.

[29] Klein, M., Rogers, G. J. & Kundur, P. A fundamental study of inter-area oscillations in power systems. *IEEE Transactions on Power Systems* **6**, 914–921 (1991).
