# Supplementary material for: Open database analysis of scaling and spatio-temporal properties of power grid frequencies
Source: Nat Commun. 2020 Dec 11;11:6362. doi: 10.1038/s41467-020-19732-7 (PMC7732984; doi:10.1038/s41467-020-19732-7)
Supplement: Supplementary file 2 — Peer Review File [file 41467_2020_19732_MOESM2_ESM.pdf]

Reviewer #2 (Remarks to the Author):

I have read the work "Open data base analysis of scaling and spatio-temporal properties of power grid Frequencies", by Gorjão and coauthors. The main message of the paper is that the analysis of the frequency fluctuations in several points (and instants) of the power grid infrastructure bears information on the underlying dynamics and it is potentially interesting for power grid planning.
In general I favour publication, for the results are original and of broad interest, covering nonlinear dynamics issues as well as practical issues. I have, though, few concerns that I suggest to consider to improve the paper.

In the first place I have noticed in several points the use of variables close to, but not perfectly adherent to, the physical quantity of interest. To make an example – a venial one admittedly – at the beginning of section "scaling ofindividual grids" p. 3, the authors declare that the whole European grid supplies hundreds of millions of inhabitants and generates 3 thousands TWh, much less than the Faroe Islands with few tens of thousands of inhabitants. The implicit message is that the two grids span several orders of magnitude, about four orders of magnitude in the population and about the same in power supplied to the grid. Still, the reader (or me, at least) feels something is missed: the unit to be compared are is the population? Or perhaps the power supplied, that might be proportionally smaller for the Faroe Islands? As the authors are comparing the findings with a scaling law for the number of nodes, quite naturally the question is: is the rough measure of four orders of magnitude correct? One (again, me at least) expects some insight opinion from the authors. This problem of quantities that are close ("proxy" is perhaps the most appropriated statistical term) becomes more acute when the authors discuss the role of the distance, that does not enter into the dynamic equation (1). Although the authors mention these difficulties in the text (e.g., in the caption of Fig. 3), I think the could be better formulated and stressed. In particular, in the section "Time to Bulk" it is mentioned that a "lower density" of nodes gives a longer effective distance respect to the "air plan routes". Actually I think the opposite: with lower distance there are less nodes per distance in eq.(1), therefore the actual distance is lower than the physical distance (imagine two points connected by a transmission line without intermediate nodes, the two points would be tightly connected even if far apart). Whatever is the right answer, it deserves more attention.

I also wish to convey some style suggestions:
1) In Fig. 3 and in the related discussion I think that a better description is that the noise saturates with the size, as it approaches the value "b" of the fit, and does not simply "decreases".
2) In the last paragraph of the first column of p. 10, the authors state that "Even using only the currently available data, there remain many open questions." I suppose it is quite the contrary: "because" the data are relatively few, many questions are unsolved: one hopes that using more data there will be less unsettled problems.
3) At the end of page 5 it is said that a probability distribution is "explained by a deterministic impact". This sounds as an oxymoron to my ear. I imagine the authors mean that excess fluctuations are determined by external drives, I suggest to improve the phrasing.
In conclusion, this work is logically sound and interesting, and deserves publication on a Journal such as Nature Communication, with few integrations.

Giovanni Filatrella

Reviewer #3 (Remarks to the Author):

The manuscript presents a detailed analysis of the power grid frequency fluctuations from an open source database created by the authors. Both the creation of the open database and the analysis are certainly of interest and relevance to a broad community of researchers, and furthermore, they pave the way to future research in many directions. The manuscript has a clear focus and it is well written. Therefore, in my opinion the manuscript should be accepted for publication. However I would like the

authors to consider the following comments:

The analysis of the noise amplitude vs population size plotted in Fig. 3 requires fitting two parameters to be compared with the scaling given in Eq. (3). I think this would more clear if Fig. 3 is plotted as $\log \epsilon$ vs the log of the population, so that the scaling (3) can be tested just looking if it decays linearly with a slope ½ .
Also, I would suggest the authors to comment on the fact that locations for which the frequency pdf has clearly a non-Gaussian shape in Fig. 2, such as FO or ES-PM, have associated a noise which fulfills the scaling law (which in principle it should hold only for Gaussian noises) even better than, for instance, US-UT whose frequency pdf is quite close to Gaussian.

There are significant differences between the kurtosis of the frequency pdf, and the kurtosis of the frequency increments Δf_{τ}. As shown in Supplementary Fig. 1, the pdf of the frequency is leptokurtic (kurtosis above three) only for four locations, and the maximum kurtosis is around 7. For most locations, kurtosis is 3 or slightly below, signaling Gaussian behavior or shorter short tails. Instead, as shown in Fig. 4, the pdf of the frequency increments is always leptokurtic, in some cases to an extreme degree (up to 300), signaling heavy tails. While I understand these two distributions correspond to different quantities and the second changes with τ, the large difference in the values of the kurtosis and different character of the tails are quite surprising and deserves some discussion.

In Fig. 2 it is quite apparent that some pdfs, such as those of FO and SE has a large degree of asymmetry which reflects in an no zero skewness as reported in supplementary table II. Contrary to kurtosis, skewness is not much discussed in the text. I would suggest the authors to consider analyzing the skewness of the frequency increments at different locations to see if approaches zero when increasing τ.

When discussing the “time to bulk”, fig. 8, authors adjust a linear behavior with respect to the distance (panel b). As discussed in the text, the linear behavior would be expected would be expected if the coupling is realized only through the shortest path, and in fact, most of the points are located outside the linear adjustment. If the bulk behavior were the outcome of a sort of diffusive process then the time would scale as the distance to the square. I suggest the authors to check if this scaling shows a better agreement than the linear one, at least for all the points except Istanbul.

In the discussion section, or in discussing Fig. 2, it may be relevant to take into account that Mallorca, despite being an island, is connected by a HVDC cable to the continental grid. While the DC connection does not provide synchrony, it helps in balancing demand and generation and thus contributes to the smaller fluctuations observed in this case as compared with other islands.

“Data selection”: It would be appropriate to specify the precise period of the data used for the different locations (it is only stated that for Gran Canaria the data is that of March 2018). It would also be appropriate to state how long is the time trace used for the correlations and for the synchronized measurement in continental Europe.

In the discussion of Figure 5 of the supplementary material, it is stated that the larger the lag, the more the increment statistics approach a Gaussian. However this is not precise for all the lines shown in the figure. Beyond $\tau=5$, for all locations except Istanbul the kurtosis remains constant or slightly moves away from 3. In fact there is one cases, Györ, for which the kurstosis systematically increases away from 3 as the time lag increases. The increasing is small and may be within the statistical error in determining the kurtosis, but nevertheless authors should consider revising the discussion of the figure.

In Supplementary Note 5, DFA, or in the main text, it would be appropriate to state the value of the

power m has been used in the analysis and why.

A few misprints and minor remarks:

In Fig. 1, Panels c)-d) include several overlapping lines which are difficult to be distinguished, for instance data from US-TX or US-UT in panel e). I would suggest to enlarge these panels or to include less lines in the same panel.

The caption of Fig. 2 states that the autocorrelation is computed for a time lag up to one hour when in panels d) - e) it is plotted up to 75 min.

In the last paragraph of the left column of page 5 it refers to “non-zero excess kurtosis” as a pointer to heavy tails of the distribution, however, since, in general, excess kurtosis can be negative, it would more appropriate to refer to “positive excess kurtosis” as a pointer to heavy tails.

In the third line of the right column of page 5, where it refers to US-TX as one of the locations for which the pdf of the increments approaches a Gaussian, authors probably mean US-UT.

In line 14 of the first paragraph of the section “Correlated dynamics within one area” where it refers to Fig. 6, authors probably mean Fig. 5.

In the last line of the right column of page 8 where it refers to “(Fig 5)”, do the authors mean Fig. 9a)?

In the x-axis of Fig. 9a), the labels “15” and “12” are too close to be distinguished from a single number.

Kurtosis is a dimensionless quantity, thus it is not clear why units of mHZ^4 are used in the vertical axis of Supplementary Figure 1. Is this a misprint?

In Supplementary note 5, DFA, the second equation has a “=” which seems out of place and it is not clear why there is a 1^S which is always 1. Also in the same equation and in the sentence after that, where it says yv,i should be $y_{v,i}$.

In Supplementary Note 6, it is stated that no inter-area oscillations can be observed between Karlsruhe and Oldenburg, which could indicate well-balanced power within Germany. While this may be the case, one should also take into account that there are only 6 modes and thus the oscillation may be there but be smaller than the intra-Hungarian oscillation, which is the one responsible of the last mode.

Replies to reviewers:

We would like to thank the reviewers for their thorough and constructive review of our work and their overall very positive judgement. Based on the comments of two reviewers, we have further enhanced the article. In particular, we have clarified the physical interpretation of certain quantities, such as total generation and population (Reviewer #2). Furthermore, we discuss the role of kurtosis in aggregated and increment statistics, the scaling of the noise as well as linear and diffusive coupling in more detail in the manuscript and the Supplementary Information (Reviewer #3).

All significant changes in the manuscript (including a new Code availability statement) are highlighted in blue color in the revised version. Below, we provide a detailed point-by-point response to the reviewer comments.

Reviewer #2 (Remarks to the Author):

I have read the work “Open data base analysis of scaling and spatio-temporal properties of power grid Frequencies”, by Gorjão and coauthors. The main message of the paper is that the analysis of the frequency fluctuations in several points (and instants) of the power grid infrastructure bears information on the underlying dynamics and it is potentially interesting for power grid planning.
In general I favour publication, for the results are original and of broad interest, covering nonlinear dynamics issues as well as practical issues. I have, though, few concerns that I suggest to consider to improve the paper.

> In the first place I have noticed in several points the use of variables close to, but not perfectly adherent to, the physical quantity of interest. To make an example – a venial one admittedly – at the beginning of section “scaling of individual grids” p. 3, the authors declare that the whole European grid supplies hundreds of millions of inhabitants and generates 3 thousands TWh, much less than the Faroe Islands with few tens of thousands of inhabitants. The implicit message is that the two grids span several orders of magnitude, about four orders of magnitude in the population and about the same in power supplied to the grid. Still, the reader (or me, at least) feels something is missed: the unit to be compared are is the population? Or perhaps the power supplied, that might be proportionally smaller for the Faroe Islands? As the authors are comparing the findings with a scaling law for the number of nodes, quite naturally the question is: is the rough measure of four orders of magnitude correct? One (again, me at least) expects some insight opinion from the authors. This problem of quantities that are close (“proxy” is perhaps the most appropriated statistical term) becomes more acute when the authors discuss the role of the distance, that does not enter into the dynamic equation (1). Although the authors mention these difficulties in the text (e.g., in the caption of Fig. 3), I think the could be better formulated and stressed.

We thank the reviewer for the observations. Indeed, there is a necessity to stress how we are using variables. In particular we should have stated explicitly that we used the population of the synchronous areas as a proxy for the number of nodes N present. As suggested by the reviewer, the term proxy is indeed the appropriate term. We thank the reviewer for pointing this out. Hence, we adjusted the caption of Fig. 3 to include: *"The population size serves as a proxy for the total generation and consumption of that area, as data on the size of the power grids is not commonly available."*

Furthermore, we paid careful attention throughout the text that physical quantities are referenced appropriately, see also next comment.

> In particular, in the section "Time to Bulk" it is mentioned that a "lower density" of nodes gives a longer effective distance respect to the "air plan routes". Actually I think the opposite: with lower distance there are less nodes per distance in eq.(1), therefore the actual distance is lower than the physical distance (imagine two points connected by a transmission line without intermediate nodes, the two points would be tightly connected even if far apart). Whatever is the right answer, it deserves more attention.

We thank the reviewer for raising this important point. Also following a suggestion by Reviewer #3, we have now redrawn Fig. 8 b) to include both a linear and a quadratic fit. The former would represent a direct connection, via the shortest path, and the latter would indicate a diffusive coupling. Based on the available data, a quadratic behaviour seems to fit better, indicating a diffusive relation. This has been included in the text in the revised paragraph(see also discussion below to the remark from Reviewer #3):
"We consider both a linear and a quadratic fit. A linear dependence is expected if the bulk behaviour is realised by coupling via the shortest available path. In contrast, if the propagation is following a diffusive pattern via multiple independent paths, we would expect a quadratic dependence of the time with respect to the distance. Indeed, the quadratic fit, following diffusive coupling, is a much better fit than a linear one, as indicated by a lower Root-Mean-Squared-Error ($RMSE$) of $RMSE(\text{squ.})=0.5$, compared to $RMSE(\text{lin.})=1.2$ seconds. Using the newly obtained fits, we find that a location only 100~km from Karlsruhe will have to independently stabilise fluctuations on the scale of 0.5 to 1 second and will then closely synchronise with the dynamics in Karlsruhe (our bulk reference). Contrary, a site 1000~km away has to stabilise already for about 3 to 5 seconds before it is fully integrated in the bulk. This gives additional guidance for the control within large synchronous areas, in particular for remote and weakly coupled sites. Clearly, these first estimates demonstrate that further research is necessary to validate and adjust spatio-temporal models of the power grid [21]"

I also wish to convey some style suggestions:

> 1) In Fig. 3 and in the related discussion I think that a better description is that the noise saturates with the size, as it approaches the value "b" of the fit, and does not simply "decreases".

We thank the reviewer for pointing out this issue. Indeed, we did not discuss the observed saturation so far. In the revised manuscript, we included the statement *"At a certain size, the noise saturates."* in the text and similarly in Fig. 3.

> 2) In the last paragraph of the first column of p. 10, the authors state that "Even using only the currently available data, there remain many open questions." I suppose it is quite the contrary: "because" the data are relatively few, many questions are unsolved: one hopes that using more data there will be less unsettled problems.

We thank the reviewer for this remark. We have corrected the text to reflect this observation. It now reads: "*Because data are still only scarcely available, there remain many open questions: [...]"*

> 3) At the end of page 5 it is said that a probability distribution is "explained by a deterministic impact". This sounds as an oxymoron to my ear. I imagine the authors mean that excess fluctuations are determined by external drives, I suggest to improve the phrasing.

We thank the reviewer for pointing out our previous statement, which combined "deterministic events" to explain a stochastic process.
In the revised manuscript, we changed the sentence as follows:
"For example in Continental Europe (DE) we observe Gaussian increments but a non-Gaussian aggregated distribution. The deviation from Gaussianity in the aggregated distribution, e.g. in terms of frequent extreme events, is likely explained by external drivers, such as market activities"

> In conclusion, this work is logically sound and interesting, and deserves publication on a Journal such as Nature Communication, with few integrations.
>
> Giovanni Filatrella

We thank Giovanni Filatrella for this positive assessment of our work.

Reviewer #3 (Remarks to the Author):

> The manuscript presents a detailed analysis of the power grid frequency fluctuations from an open source database created by the authors. Both the creation of the open database and the analysis are certainly of interest and relevance to a broad community of researchers, and furthermore, they pave the way to future research in many directions. The manuscript has a clear focus and it is well written. Therefore, in my opinion the manuscript should be accepted for publication. However I would like the authors to consider the following comments:

The analysis of the noise amplitude vs population size plotted in Fig. 3 requires fitting two parameters to be compared with the scaling given in Eq. (3). I think this would more clear if Fig. 3 is plotted as $\log \epsilon$ vs the log of the population, so that the scaling (3) can be tested just looking if it decays linearly with a slope ½ .

We thank the reviewer for the suggestion of a double logarithmic scale. Unfortunately, re-drawing Fig. 3 in a double logarithmic scale does not allow easy estimation of the necessary parameters. Suppose the function is given as $\varepsilon = a/\sqrt[c]{N} + b$, as we conjecture. In this case, applying the logarithm yields $log(\varepsilon) = log(a/\sqrt[c]{N} + b)$. Unfortunately, b>0, as also pointed out by Reviewer #2, and hence we cannot use $log(\varepsilon) = log(a) - 1/c \times log(N)$, rendering it impractical to fit the parameters in a double logarithmic scale.

Also, I would suggest the authors to comment on the fact that locations for which the frequency pdf has clearly a non-Gaussian shape in Fig. 2, such as FO or ES-PM, have associated a noise which fulfills the scaling law (which in principle it should hold only for Gaussian noises) even better than, for instance, US-UT whose frequency pdf is quite close to Gaussian.

We thank the reviewer for the comment. Indeed, this seemingly counterintuitive effect was not addressed, yet we offer a conclusive answer for the observed behaviour at this stage of our research. Hence, we included the following statement:
"Interestingly, while Faroe Islands (FO) and Mallorca (ES-PM) do display non-Gaussian probability density functions, they follow the proposed scaling law. Why this is the case and how a fully non-Gaussian scaling law could capture this even better remain open questions for future work."

There are significant differences between the kurtosis of the frequency pdf, and the kurtosis of the frequency increments Δf_{τ}. As shown in Supplementary Fig. 1, the pdf of the frequency is leptokurtic (kurtosis above three) only for four locations, and the maximum kurtosis is around 7. For most locations, kurtosis is 3 or slightly below, signaling Gaussian behavior or shorter short tails. Instead, as shown in Fig. 4, the pdf of the frequency increments is always leptokurtic, in some cases to an extreme degree (up to 300), signaling heavy tails. While I understand these two distributions correspond to different quantities and the second changes with τ, the large difference in the values of the kurtosis and different character of the tails are quite surprising and deserves some discussion.

This is indeed a very good observation. Due to the limited space in the main text, we included a discussion comparing the role of a high kurtosis of both the increment and the aggregated statistics in the Supplementary Information and included the following in the main text:
"We further analyse the differences between aggregated kurtosis and increment kurtosis in Supplementary Note 1"

In Fig. 2 it is quite apparent that some pdfs, such as those of FO and SE has a large degree of asymmetry which reflects in an no zero skewness as reported in supplementary table II. Contrary to kurtosis, skewness is not much discussed in the text. I would suggest the authors to consider analyzing the skewness of the frequency increments at different locations to see if approaches zero when increasing τ.

We thank the reviewer for the observation and suggestion. When analysing the scaling with size or the increment kurtosis as a function of the time lag, we can rely on previous work or other literature. In contrast, to our knowledge, the non-vanishing skewness in various synchronous areas and in their increment statistics has not been studied in depth. Hence, we refrained from going into too much detail here and have to work on a joint data analysis, statistical modelling and engineering perspective to explain the role of skewness in more detail in the future. To signal this to the reader, we have included the following in the outlook: *"From a statistical modelling perspective, it would be interesting to investigate the scaling of higher moments, i.e. skewness and kurtosis, with time lag and size in more detail."*

When discussing the "time to bulk", fig. 8, authors adjust a linear behavior with respect to the distance (panel b). As discussed in the text, the linear behavior would be expected would be expected if the coupling is realized only through the shortest path, and in fact, most of the points are located outside the linear adjustment. If the bulk behavior were the outcome of a sort of diffusive process then the time would scale as the distance to the square. I suggest the authors to check if this scaling shows a better agreement than the linear one, at least for all the points except Istanbul.

This is another excellent suggestion for which we are very thankful. Indeed, the diffusive coupling is a much better description than the linear coupling as we show in the revised Fig. 8 and also by comparing the root-mean-squared-error (RMSE) in the text. We discuss the role of linear compared to diffusive coupling in the text within the revised paragraph discussing Fig. 8 as follows:
"We consider both a linear and a quadratic fit. A linear dependence is expected if the bulk behaviour is realised by coupling via the shortest available path. In contrast, if the propagation is following a diffusive pattern via multiple independent paths, we would expect a quadratic dependence of the time with respect to the distance. Indeed, the quadratic fit, following diffusive coupling, is a much better fit than a linear one, as indicated by a lower Root-Mean-Squared-Error 0.5, compared to 1.2 seconds in the linear case. Using the newly obtained fits, we find that a location only 100~km from Karlsruhe will have to independently stabilise fluctuations on the scale of 0.5 to 1 second and will then closely synchronise with the dynamics in Karlsruhe (our bulk reference). Contrary, a site 1000~km away has to stabilise already for about 3 to 5 seconds before it is fully integrated in the bulk. This gives additional guidance for the control within large synchronous areas, in particular for remote and weakly coupled sites. Clearly, these first estimates demonstrate that further research is necessary to validate and adjust spatio-temporal models of the power grid [21]"

In the discussion section, or in discussing Fig. 2, it may be relevant to take into account that Mallorca, despite being an island, is connected by a HVDC cable to the continental grid. While the DC connection does not provide synchrony, it helps in balancing demand and generation and thus contributes to the smaller fluctuations observed in this case as compared with other islands.

This is an important point raised by the reviewer. We have included a brief discussion of the role of HVDC in general and specifically their role for Mallorca, where it could help to reduce the large deviations observed in Iceland and Faroe Islands as follows:
"Note that some of the synchronous areas considered here are indeed coupled via high-voltage direct current (HVDC) lines but still possess independent synchronous behavior. Specifically, the British (GB), Continental (DE), Baltic (EE) and Nordic (SE) European areas as well as Mallorca are connectedd in this way. The HVDC connection of Mallorca towards Continental Europe might be the reason it displays overall smaller deviations than FO or IS, which cannot access another large synchronous area for balance."

"Data selection": It would be appropriate to specify the precise period of the data used for the different locations (it is only stated that for Gran Canaria the data is that of March 2018). It would also be appropriate to state how long is the time trace used for the correlations and for the synchronized measurement in continental Europe.

As we pointed out in the first sentence of this subsection, a detailed overview of the available data is presented in another document. Still, we included the following brief statement to give a better overview of the data:
"This data set contains recordings of twelve independent synchronous regions recorded between 2017 and 2020. While some locations, such as the Faroe Islands only contain a single week of data, other regions, such as Continental Europe have been monitored for several months or years, for more details see [Jumar et al]."

In the discussion of Figure 5 of the supplementary material, it is stated that the larger the lag, the more the increment statistics approach a Gaussian. However this is not precise for all the lines shown in the figure. Beyond $\tau=5$, for all locations except Istanbul the kurtosis remains constant or slightly moves away from 3. In fact there is one cases, Györ, for which the kurstosis systematically increases away from 3 as the time lag increases. The increasing is small and may be within the statistical error in determining the kurtosis, but nevertheless authors should consider revising the discussion of the figure.

We agree that our previous statement was imprecise. Hence, we updated the discussion by changing the sentence to *"Computing the kurtosis κ of the increment statistics Δf_τ at different lags τ, shows that the deviations from the Gaussian ($\kappa^\text{Gaussian}=3$) decrease on average. While the kurtosis shows a small increase in Győr with increasing time lag, the kurtosis at Istanbul and Lisbon is substantially reduced, see Supplementary Fig.5."*

In Supplementary Note 5, DFA, or in the main text, it would be appropriate to state the value of the power m has been used in the analysis and why.

We have added the polynomial order of fitting, *m=1*, in agreement with a recent publication (Ref. Meyer, P. G., Anvari, M. & Kantz, H. In Chaos 30, 013130 (2020)). We added in the caption of Fig. 7 the following: *"[...] with order $m=1$, in accordance with Ref.[52], [...]"*

A few misprints and minor remarks:

In Fig. 1, Panels c)-d) include several overlapping lines which are difficult to be distinguished, for instance data from US-TX or US-UT in panel e). I would suggest to enlarge these panels or to include less lines in the same panel.

We thank the reviewer for the suggestions. Panels c-e in Fig. 1 mainly serve an illustrative purpose. We improved the readability by changing the order in which the lines were added and introduced a small offset in panels c-e.

The caption of Fig. 2 states that the autocorrelation is computed for a time lag up to one hour when in panels d) - e) it is plotted up to 75 min.

We fixed this typo and state in caption of Fig. 2: *"We compute the autocorrelation of each area for a time lag of up to 75 mins."*

In the last paragraph of the left column of page 5 it refers to "non-zero excess kurtosis" as a pointer to heavy tails of the distribution, however, since, in general, excess kurtosis can be negative, it would more appropriate to refer to "positive excess kurtosis" as a pointer to heavy tails.

We followed the suggestion and now call the term "positive excess kurtosis".

In the third line of the right column of page 5, where it refers to US-TX as one of the locations for which the pdf of the increments approaches a Gaussian, authors probably mean US-UT.

We thank the reviewer for pointing out this typo, which we corrected.

In line 14 of the first paragraph of the section "Correlated dynamics within one area" where it refers to Fig. 6, authors probably mean Fig. 5.

Indeed, the reference to Fig. 6 is intentional. To make this change of subject a bit more clear, we included an additional line break.

In the last line of the right column of page 8 where it refers to "(Fig 5)", do the authors mean Fig. 9a)?

The reference to Fig. 5 is intentional, yet we forgot to include “cf.” whilst referring to the figure, in order to guide the reader to re-examine Fig. 5, where one can notice the variations between the 4 different measurement sites.

> In the x-axis of Fig. 9a), the labels “15” and “12” are too close to be distinguished from a single number.

The spacing has been adopted by changing the position of the x-ticks.

> Kurtosis is a dimensionless quantity, thus it is not clear why units of mHZ^4 are used in the vertical axis of Supplementary Figure 1. Is this a misprint?

This was a misprint and has been corrected.

> In Supplementary note 5, DFA, the second equation has a “=” which seems out of place and it is not clear why there is a 1^S which is always 1. Also in the same equation and in the sentence after that, where it says yv,i should be $y_{v,i}$.

We thank the reviewer for pointing out these typos. We were missing “_” in the latex commands, which caused some confusion in the equation.

> In Supplementary Note 6, it is stated that no inter-area oscillations can be observed between Karlsruhe and Oldenburg, which could indicate well-balanced power within Germany. While this may be the case, one should also take into account that there are only 6 modes and thus the oscillation may be there but be smaller than the intra-Hungarian oscillation, which is the one responsible of the last mode.

We included the limitation of only 6 modes in Supplementary Note 6.

The thank the reviewer for the thorough examination and very valuable input and suggestions to the manuscript and supplemental material.

Reviewer #2 (Remarks to the Author):

The authors have answere my objections. I suggest pubblication.

Reviewer #3 (Remarks to the Author):

As indicated in the first report the manuscript has a clear focus and it is of great interest and relevance to a broad community of researchers and paves the way to future research in several directions. In this revised version, authors have properly addressed the remarks raised in the initial report. Therefore I fully recommend the manuscript to be accepted for publication in its present form.
